# The Small GTPase RAC1/CED-10 Is Essential in Maintaining Dopaminergic Neuron Function and Survival Against α-Synuclein-Induced Toxicity

**DOI:** 10.1007/s12035-018-0881-7

**Published:** 2018-02-10

**Authors:** Hanna Kim, Carles Calatayud, Sanjib Guha, Irene Fernández-Carasa, Laura Berkowitz, Iria Carballo-Carbajal, Mario Ezquerra, Rubén Fernández-Santiago, Pankaj Kapahi, Ángel Raya, Antonio Miranda-Vizuete, Jose Miguel Lizcano, Miquel Vila, Kim A. Caldwell, Guy A. Caldwell, Antonella Consiglio, Esther Dalfo

**Affiliations:** 1 Department of Biological Sciences, The University of Alabama, Tuscaloosa, AL 35487 USA; 2 Department of Pathology and Experimental Therapeutics, Bellvitge University Hospital-IDIBELL, 08028 L’Hospitalet de Llobregat, Spain; 3 Institute of Biomedicine of the University of Barcelona (IBUB), Barcelona, 08908 Spain; 4 Center of Regenerative Medicine in Barcelona (CMRB), Center for Networked Biomedical Research on Bioengineering, Biomaterials and Nanomedicine (CIBER-BBN), Hospital Duran i Reynals, 08908 L’Hospitalet de Llobregat, Spain; 5 Buck Institute for Research on Aging, 8001 Redwood Boulevard, Novato, CA 94945 USA; 6 Neurodegenerative Diseases Research Group, Vall d’Hebron Research Institute-Center for Networked Biomedical Research on Neurodegenerative Diseases (CIBERNED), 08035 Barcelona, Spain; 7 Laboratory of Parkinson Disease and Other Neurodegenerative Movement Disorders, Department of Neurology: Clinical and Experimental Research, IDIBAPS – Hospital Clínic de Barcelona, 08036 Barcelona, Spain; 8 Catalan Institution for Research and Advanced Studies (ICREA), 08010 Barcelona, Spain; 9 Instituto de Biomedicina de Sevilla, Hospital Universitario Virgen del Rocío/CSIC/ Universidad de Sevilla, 41013 Sevilla, Spain; 10 Department of Biochemistry and Molecular Biology, Institut de Neurociències, Faculty of Medicine, M2, Universitat Autònoma de Barcelona (UAB), Bellaterra Campus, Cerdanyola del Vallés, Barcelona, Spain; 11 Department of Molecular and Translational Medicine, University of Brescia, Brescia, Spain; 12 Faculty of Medicine, University of Vic-Central University of Catalonia (UVic-UCC), Can Baumann, 08500 Vic, Spain

**Keywords:** Parkinson’s disease, Dopaminergic neurons, Alpha-synuclein accumulation, Autophagy impairment, *RAC1/ced-10*

## Abstract

Parkinson’s disease is associated with intracellular α-synuclein accumulation and ventral midbrain dopaminergic neuronal death in the *Substantia Nigra* of brain patients. The Rho GTPase pathway, mainly linking surface receptors to the organization of the actin and microtubule cytoskeletons, has been suggested to participate to Parkinson’s disease pathogenesis. Nevertheless, its exact contribution remains obscure. To unveil the participation of the Rho GTPase family to the molecular pathogenesis of Parkinson’s disease, we first used *C elegans* to demonstrate the role of the small GTPase *RAC1* (*ced-10* in the worm) in maintaining dopaminergic function and survival in the presence of alpha-synuclein. In addition, *ced-10* mutant worms determined an increase of alpha-synuclein inclusions in comparison to control worms as well as an increase in autophagic vesicles. We then used a human neuroblastoma cells (M17) stably over-expressing alpha-synuclein and found that *RAC1* function decreased the amount of amyloidogenic alpha-synuclein. Further, by using dopaminergic neurons derived from patients of familial LRRK2-Parkinson’s disease we report that human RAC1 activity is essential in the regulation of dopaminergic cell death, alpha-synuclein accumulation, participates in neurite arborization and modulates autophagy. Thus, we determined for the first time that *RAC1/ced-10* participates in Parkinson’s disease associated pathogenesis and established *RAC1/ced-10* as a new candidate for further investigation of Parkinson’s disease associated mechanisms, mainly focused on dopaminergic function and survival against α-synuclein-induced toxicity.

**Electronic supplementary material:**

The online version of this article (10.1007/s12035-018-0881-7) contains supplementary material, which is available to authorized users.

## Introduction

Parkinson’s disease (PD) is the second most frequent neurodegenerative disorder in the elderly. While most of cases are sporadic, monogenic PD caused by pathogenic point mutations in PD-associated genes occurs in less than 10% of cases (reviewed in [1]). The common neuropathological hallmarks of PD include a selective loss of the dopaminergic neurons (DAn) in the *Substantia Nigra pars compacta* and aggregation of the protein alpha-synuclein (α-SYN) in the surviving DAn and in the so called Lewy bodies (LB) and Lewy neurites (LN) which are found in the few surviving DAn (reviewed in [2]). α-SYN is intrinsically misfolded in pathological conditions such as PD [3] and forms multiple conformations, including amyloidogenic oligomers [4, 5] implicated in α-SYN toxicity [6].

There exist evidences of an essential role of actin cytoskeleton disruptions in both DAn cell death [7, 8] and α-SYN accumulation [9]. In fact, the cytoskeleton is an important target of α-SYN [10] and neuronal microtubule-kinesin function could be impaired by α-SYN oligomers [11]. Actin cytoskeletal organization is regulated by small GTPases of the Rho family encompassing Rho, Cdc42 and Rac subfamily members [7]. These proteins act as molecular switches as they alternate between the active GTP-bound and the inactive GDP-bound forms [8, 16]. GTP binding increases the activity, and the hydrolysis of GTP to GDP renders the protein inactive. More specifically, RAC1 activity is mainly associated with cellular processes involving the regulation of actin polymerization such as cell migration, lamellipodia extension or the phagocytosis of dead cells or engulfment [12]. In addition, RAC1 participates in the extension and retraction of neurites [13] and, together with other members of the Rho family, govern changes in neuronal morphology and the dynamics of neuronal processes (reviewed in [8]).

RAC1 function has been associated with two PD-related genes. We have previously shown in *C elegans* that RAC1 is ubiquitylated by PARKIN [14], mutated in the juvenile variant of PD. Likewise, Leucine-rich repeat kinase 2 (LRRK2), in which mutations cause the most common form of familial PD [15], strongly and selectively binds to RAC1 [16]. Furthermore, neuronal apoptosis induced in DAn in vitro is correlated with decreased RAC1 activity [17]. In contrast, in a monkey model of PD, it was suggested that aberrant activation of RAC1 in microglia may contribute to enhanced production of ROS underlying the death of neighboring DAn [18]. Therefore understanding the cytoskeletal mechanisms associated with DA cell death and α-SYN degradation is important to elucidate other causative agents of the PD pathophysiology.

Autophagic flux is profoundly disrupted in PD patients (reviewed in [1] and α-SYN is normally degraded by autophagy [19]. Indeed, autophagy has been associated with PD pathogenesis through several genes, such as *LRRK2* [20], *ATG9A* [21] or *ATG8/LC3* [22], and cellular processes such as lysosomal disruption [23, 24]. In addition, autophagy-related gene products are required for apoptotic clearance, either in dying cells or through a role in engulfment, in where *RAC1* has a pivotal role [25–27].

In the present study we have systematically investigated *RAC1* function in three disease models of PD including the following: (a) *C elegans* models of PD; (b) human-derived neuroblastoma BE(2) (M17) cells stably over-expressing α-SYN, wherein amyloidigenic accumulation of α-SYN is induced by sodium butyrate; and (c) iPSC-derived DAn generated by cell reprogramming of somatic skin cells from patients with monogenic LRRK2-associated PD [20]. Using these models, we determine for the first time that *RAC1/ced-10* participates specifically in PD-associated pathogenesis and establish *RAC1/ced-10* as a new candidate to be considered for the investigation of PD-associated mechanisms, mainly focused on DA function and survival against α-SYN-induced toxicity.

## Results

### *RAC1/ced-10* Cell-Autonomous Depletion in DAn Hampers Dopamine- Associated Behavior in the Presence of α-SYN and Accelerates α-SYN Induced DAn Death in *C elegans*

We first investigated the role of *RAC1/ced-10* in DAn function, by performing behavioral assays through analyzing the DA behavior in *ced-10(n3246)* mutant animals. The mutation *ced-10(n3246)* is a G-to-A transition resulting in a change of glycine 60 of CED-10 to arginine (G60R) which results in non-null altered function [28, 29]. The severity of this allele is stronger in contrast to other *ced-10* alleles (11). To explore the role of *ced-10* in PD pathogenesis, all the experiments included in this study involving the *ced-10* gene were performed in a *ced-10(n3246)* mutant background. To simplify, *ced-10(n3246)* is named *ced-10* from here on.

The basal slowing response is a DA dependent behavior widely used in *C elegans* for analyzing the functionality of the DA system [30–32]. Briefly, worms decrease locomotion speed when in physical contact with a food source whereas the turn frequency increases when worms leave the food source [31, 33]. The *cat-2* gene encodes the enzyme tyrosine hydroxylase required for the synthesis of dopamine. Accordingly, *cat-2(e1112)* mutant worms have decreased levels of dopamine and altered DA behavior [30, 33] and were used as positive control.

**Fig. 1:**
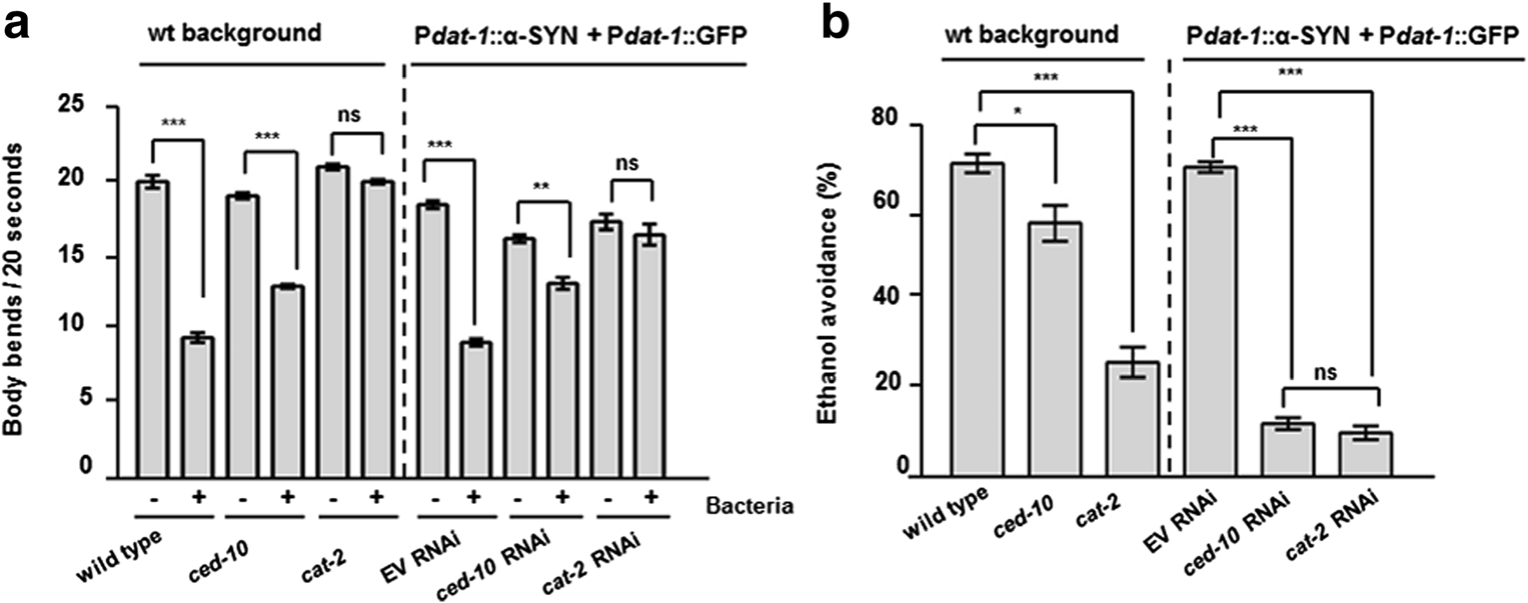
DAn function is hampered by specific depletion of *RAC1/ced-10* in DAn expressing α-SYN in *C elegans*, **a** Modulation of the locomotor rate. Well-fed animals were transferred to assay plates without or with a bacterial lawn (−/+) and 5 min later, the locomotor rate (body bends every 20 s) of each animal was analyzed. Statistical significance shows comparison of the bending within same genotype animals (wild type, *ced-10(n3246)* and *cat-2 (e1112)*) without (−) or with (+) bacteria. RNAi experiments indicate P*dat-1*::α-SYN *+*P*dat-1*:: GFP worms fed with EV (empty vector) or with the indicated RNAi clones. **b** Test of ethanol avoidance. The percentage (%) of ethanol avoidance was analyzed at the indicated genotypes. Statistical analysis shows comparisons between wild type animals and mutants, and P*dat-1*::α-SYN *+*P*dat-1*:: GFP worms fed either with EV (empty vector) or with the indicated RNAi clones. *cat-2 (e1112)* mutant worms were included as positive controls for both assays. Slow response assay and ethanol avoidance behaviors were hampered in *ced-10* depleted animals and not in *ced-10(n3246)* mutants. Data are mean ± SEM. * P < 0.05, **P < 0.01, ***P < 0.001, *ns* non-significant. Statistics: one-way ANOVA, Tukey post hoc test for multiple comparisons. Between 20 and 30 worms were used in three independent replicates

The locomotion speed of the nematodes, represented by the body bends every 20 s, was measured in the absence/presence of bacteria (+/−) (Fig. 1a). In a wild type background (wt background), both wild type and *ced-10* animals decreased the locomotion speed in the presence of food, thus showing unaltered basal slowing response. In contrast, in *cat-2(e1112)* mutants the locomotion speed was not significantly decreased by the presence of food (Fig. 1a wt background, and Table 1). Similarly to the slow basal response, avoidance against ethanol is a sensory behavior associated with DA signaling [34]. A slight decrease was observed in the ethanol avoidance test performed in *ced-10* mutant animals in a wt background (Fig. 1b wt background and Table 2).

**Table 1:** Slow dose response quantifications

Genotype	W/o food (body bends/20 s)	With food (body bends/20 s)	Significance (*P* value)
Wild type	20.00 ± 0.423	9.55 ± 0.328	*P* < 0.001
*ced-10(n3246)*	19.05 ± 0.211	13.00 ± 0.145	*P* < 0.001
*cat-2 (e1112)*	21.00 ± 0.191	20.00 ± 0.162	ns
UA196 on EV	18.40 ± 0.255	9.20 ± 0.257	*P* < 0.001
UA196 on *ced-10* RNAi	16.00 ± 0.254	13.15 ± 0.392	*P* < 0.001
UA196 on *cat-2* RNAi	17.25 ± 0.502	16.40 ± 0.689	ns

Mean and SEM reported, *n* = 20. One-way ANOVA

**Table 2:** Ethanol avoidance quantifications

Genotype	Avoidance (% of worms)	*N*	Significance (*P* value)
Wild type	71.21 ± 2.120	155	–
*ced-10(n3246)*	57.80 ± 3.957	94	*P* < 0.05
*cat-2 (e1112)*	26.67 ± 3.335	80	*P* < 0.001
UA196 on EV	70.35 ± 1.213	122	*–*
UA196 on *ced-10* RNAi	12.95 ± 1.328	95	*P* < 0.001
UA196 on *cat-2* RNAi	10.902 ± 1.524	175	*P* < 0.001

Mean and SEM reported. One-way ANOVA

The absence of significant dopaminergic behavioral alterations observed in *ced-10* mutants might be the consequence of compensating mechanisms existing in the worm that mask the effect of *ced-10* function specifically in DAn. To discard any effect of *ced-10* function onto DA responses, *ced-10* was depleted conditionally in DAn by RNAi, in a *C. elegans* PD model in which DAn undergo age-dependent neurodegeneration following human *α*-SYN overexpression [35]. In this model, animals express both α-SYN and GFP in DAn. Importantly, this is a neuronal-sensitive RNAi strain whereby the impact of RNAi knockdown targeting gene candidates can be selectively examined exclusively in the DAn [36] To simplify, this strain is called P*dat-1*::α-SYN + P*dat-1*::GFP herein. Animals exposed to *ced-10* RNAi showed a mild but significant altered slow response assay (Fig. 1a and Table 1). In addition, selective depletion of *ced-10* in DAn resulted in similar avoidance against ethanol as *cat-2* depleted animals in the ethanol avoidance test (Fig. 1b and Table 2). Therefore, in the presence of α-SYN, *ced-10* function is specifically necessary in *C elegans* DAn to execute a correct DA behavioral response.

**Fig. 2:**
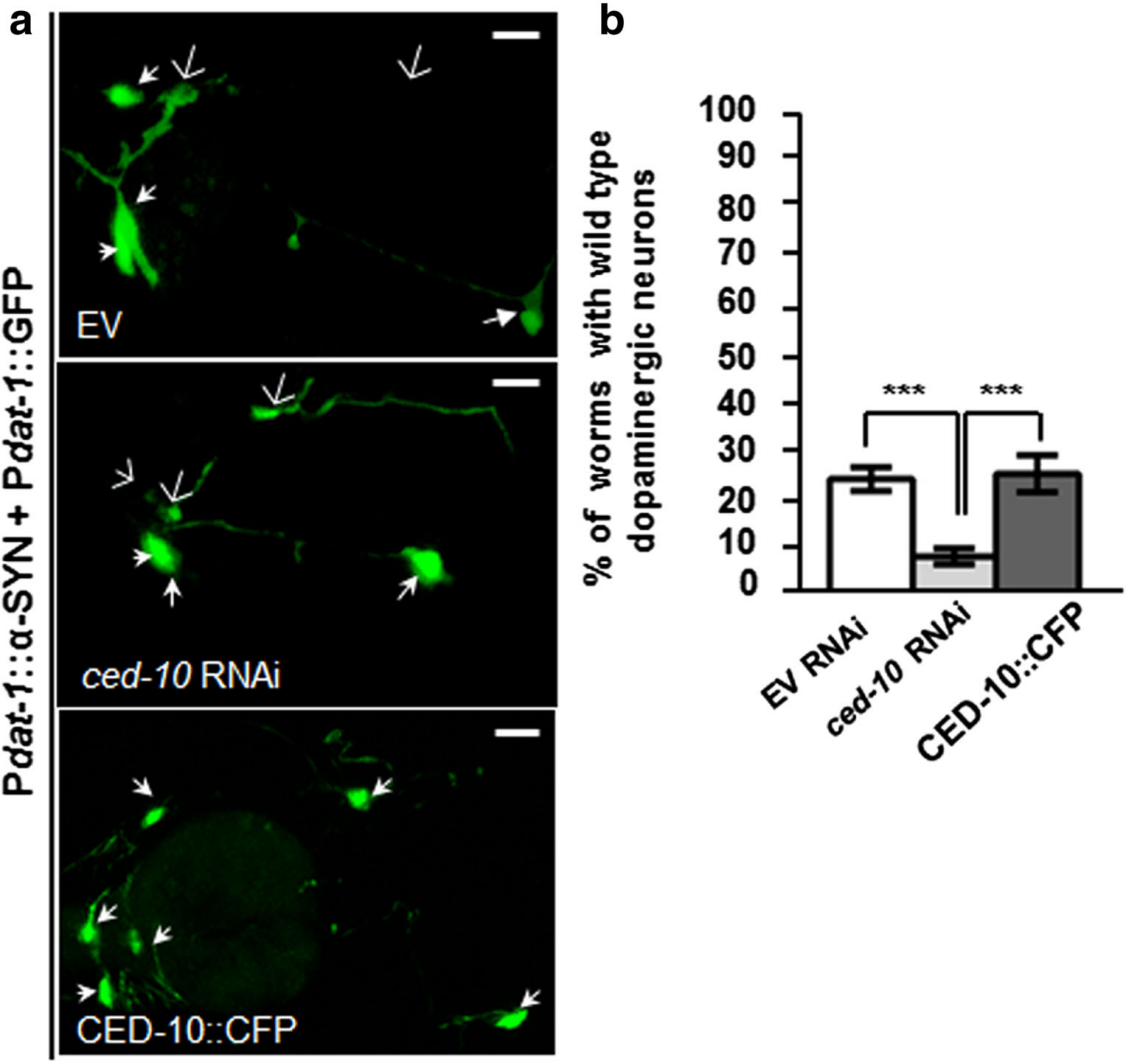
CED-10 protects DAn from α-SYN-induced DA cell death in *C elegans*. Representative RNAi empty vector (EV) fed worms expressing GFP and α-SYN specifically in DAn (P*dat-1*:: α-SYN + P*dat-1*:: GFP) at L4 + 7days (9 days post hatching) and fed with empty vector (EV) or *ced-10* RNAi clones. Filled white arrowhead labels healthy neurons whereas degenerated or missing neurons are labeled with an open arrow. **a** *ced-10* depletion reduces the amount of DAn per worm and the expression of the CED-10::CFP transgene {*baEx167* [P*ced-10*::CFP::*ced-10*]} delays the DA cell death at the same age. Magnification bar is 30 μm. **b** Percentage of P*dat-1*:: α-SYN + P*dat-1*:: GFP worms non-depleted and *ced-10* depleted by RNAi, that had the full complement of six anterior DAn at day 7 post L4. The transgenic derivative strain UA281 expressing CFP::CED-10 (CED-10 wild type), carrying the array *baEx167* [P*ced-10*::CFP*::ced-10*] ameliorates the DA cell death. Data are mean ± SEM. Statistics were obtained by comparing *ced-10* RNAi depleted worms or worms containing the CFP::CED-10 array with the corresponding EV fed animals. Statistics: ***P < 0.001, one-way ANOVA, Tukey’s post hoc test. Number of animals is 30–35 per condition, and the experiment was repeated three times independently

This negative impact of *ced-10* depletion on DAn behavior brought us to explore the relevance of *ced-10* deficiency in DAn cell death in the above mentioned strain P*dat-1*::α-SYN + P*dat-1*::GFP_*.*_ There exist four pairs of DAn in *C elegans* hermaphrodites, three of them (CEPD, CEPV and ADE) located in the anterior part, and one pair, the PDE, located in the posterior part of the nematode [37]. In this nematode, when human α-SYN is expressed in DAn, the six DAn within the anterior region of the worm display progressive degenerative characteristics [38]. To draw parallels between human PD evidenced in aged populations and this worm model, we sought to determine the relevance of *ced-10* depletion at 9 days (L4 + 7) post hatching. Cell bodies and neuronal processes were assessed to determine whether these structures were normal or displayed degenerative changes, and consequently considered wild type neurons (Fig. 2). After feeding worms with EV RNAi, 24.93 ± 2.54% of animals showed the six anterior wild type DAn. In contrast, *ced-10* RNAi knockdown significantly enhanced DA neurodegeneration, 8.33 ± 1.67% of animals showed the intact set of DAn (***P < 0.001)) in comparison with EV control (Fig. 2a, b). Animals expressing the fusion protein, CFP::CED-10, under *ced-10* promoter, rescued α-SYN induced-neurodegeneration (***P < 0.001) (Fig. 2a, b). We found that neurodegeneration was accelerated already at day 3 and 5 (L4 + 1 and L4 + 3, respectively) post hatching (Online Resource 1) thus corroborating the impact of *ced-10* in α-SYN-induced DA cell death at younger ages.

### CED-10 Expression Decreases α-SYN Inclusions Formation in *C elegans*

**Fig. 3:**
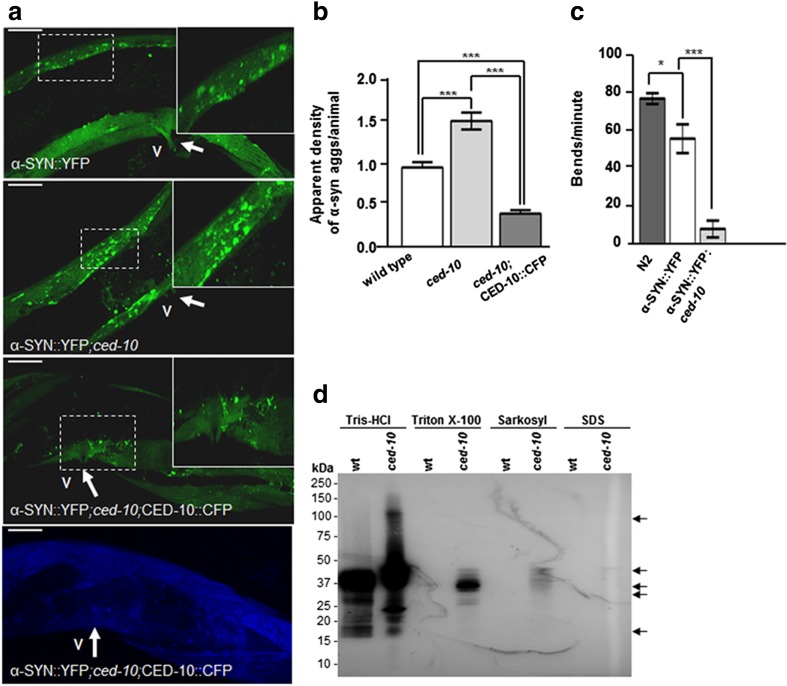
*ced-10* decreases α-SYN inclusions in *C elegans*. **a** Representative confocal pictures obtained from animals containing the genomic array *pkIs2386* [*Punc-54*_*::*_α-SYN*::*YFP] expressing α-SYN in body wall muscle cells at L4 + 5 days of development (7 days post hatching). Green staining in all figures represents α-SYN::YFP inclusions in muscle cells. The vulva (V, thick arrow) was used as a reference to analyze the same central section in all worms. A representative area was highlighted and expanded in each panel, to better visualize the α-SYN::YFP accumulation. **a** (first panel) α-SYN inclusions were detected in a *C. elegans* model of α-SYN miss folding in which α-SYN is expressed under the control of the *unc-54* promoter. **a** (second panel) α-SYN apparent aggregates are increased in *ced-10*(*n3246*) mutant nematodes. **a** (third panel) CFP::CED-10 expression (array *baEx167* [P*ced-10*::CFP::CED-10]) decreased the number of α-SYN inclusions in a *ced-10*(*n3246*) background. **a** (fourth panel) The blue fluorescence marker (CFP) represented the endogenous expression of CED-10 in a *ced-10*(*n3246*) background for rescuing α-SYN accumulation. Magnification bar is 10 μm. **b** Quantification of the number of α-SYN inclusions per area. Data are mean ± SEM. Between 30 and 35 animals were analyzed per genotype. Three different transgenic lines expressing CFP::CED-10 were generated and analyzed independently. Statistics: one-way ANOVA with a Tukey post hoc test. ***P < 0.005. **c** The movement of YFP::α-SYN animals is hampered by the mutation *ced-10n(3246)*. Thrashing behavior (bends/min) was video recorded and the resulting images were analyzed by the ImageJ software. Data are mean ± SEM. Between 20 and 30 animals were recorded per experiment and the same experiment was repeated 3 independent times. **d** Immunoblotting analysis of protein extracts from 5 days post L4 old YFP::α-SYN synchronized animals, using anti-α- SYN antibody without and with the *ced-10(n3246)* mutation (wild type and *ced-10* respectively). The amount of α-SYN insoluble species was increased by the *ced-10(n3246)* mutation.

The term phagocytosis refers also to the mechanism by which certain cells engulf and digest other cells and also larger particles or even anomalous inclusions or aggregates [39, 40]. *RAC1/ced-10* is the converging gene of the engulfment machinery mobilizing actin pseudopodia in phagocytic cells [12]. Therefore, we considered the possibility of *ced-10* playing a role in the clearance/phagocytosis of α-SYN inclusions. We used a nematode model of PD, in which human α-SYN is fused to the yellow fluorescent protein (YFP) under control of the body wall muscle *unc-54* promoter, transgene *pkIs2386* [P*unc-54*::*α-SYN*::*YFP*] [41]. With this approach we examined changes in apparent aggregate density or aggregate count of pathogenic α-SYN conjugated to fluorescent YFP in muscle cells [42], without considering neuronal side effects. Accordingly, *ced-10* animals were crossed with *pkIs2386* worms and the number of α-SYN aggregates was evaluated in aged worms at 7 days post hatching. This *ced-10* mutation increased to 1.5 units the apparent density of α-SYN inclusions in comparison to control worms (0.9 ± 0.06 vs. 1.49 ± 0.06, respectively; ***P < 0.001) (Fig. 3a, b) thus suggesting a deleterious effect of the *ced-10* mutation in the generation of α-SYN aggregates. Importantly, the increase in α-SYN apparent aggregates was abolished in transgenic *ced-10* mutants expressing the CFP::CED-10 fusion protein (array *baEx167[*P*ced-10*::CFP::*ced-10]*) (0.42 ± 0.03 in worms expressing CED-10 wild type vs. 1.49 ± 0.06 in *ced-10* mutant worms respectively and Fig. 3a, b, ***P < 0.001), showing that the lack of *ced-10* is contributing to α-SYN accumulation.

The number of body thrashes or thrashing have been used extensively to identify modifiers of protein aggregation [41, 43]. Thrashing in *C elegans* can be measured in liquid media by counting the number of body bends per unit of time [44]. Using this method, we confirmed the observed damaged motility (Fig. 3c and **Online Resources** **2****–****4**) of the PD worms in a *ced-10* background. Whereas a decrease of 27% in the bending are observed in animals expressing YFP::α-SYN in comparison to the wild type N2 wild type strain (55.01 ± 7.5 vs. 75.96 ± 2.8 bends/min, respectively), the number of bends decreases almost 90% in animals harboring the *ced-10* mutation in a YFP::SYN background in comparison with N2 wild type animals (7.93 ± 4.4 vs. 75.96 ± 2.8 bends/min, respectively) and 70% in comparison with worms expressing YFP::SYN, without the *ced-10* mutation (7.93 ± 4.4 vs. 55.01 ± 7.5 bends/min, respectively). Thus, increased α-SYN in muscle with its concomitant locomotion decrease in *ced-10* mutants, indicate the involvement of *RAC1/ced-10* in the process of α-SYN accumulation in *C. elegans*.

α-SYN variants that form oligomers and protofibrils are associated to the most severe DAn nigral loss in PD models [1, 6]. To identify the biochemical nature of the apparent α-SYN aggregates increased by this *ced-10* mutation, worm lysates from *pkIs2386* worms without and with the *ced-10* mutation at L4+ 5 days of development, were sequentially extracted by detergent-containing buffers [45] and the amount of α-SYN extracted in each fraction was assessed by immunoblotting (Fig. 3d). A faint band of 19 kDa was detected in the Tris-HCl fraction, most probably corresponding to the α-SYN monomer staining*.* The number of oligomeric species is increased in *ced-10* mutant animals within all analyzed fractions.

### Autophagy Is Impaired in *ced-10(n3246)* Mutant Animals

**Fig. 4:**
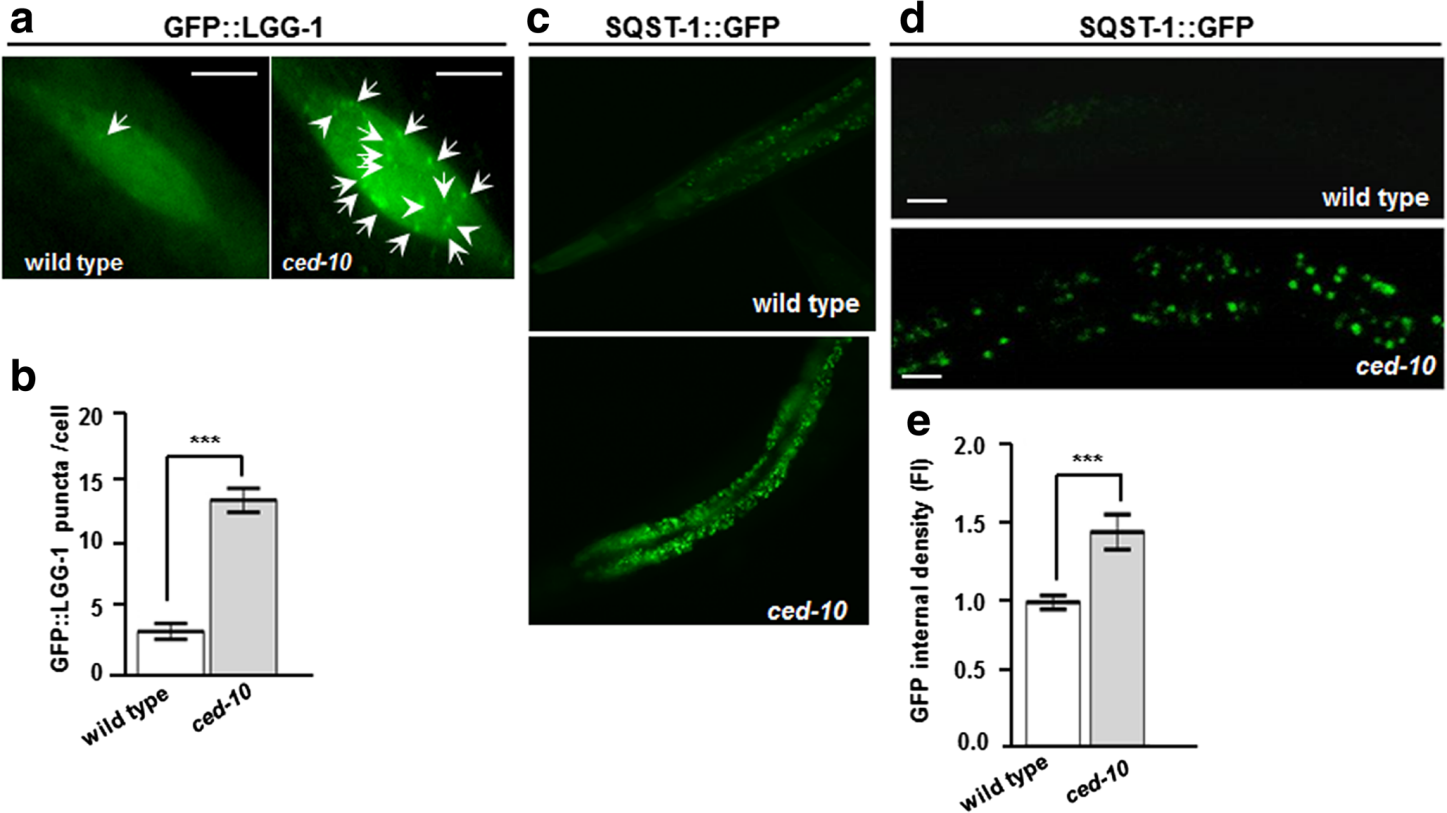
Autophagic markers accumulate in *ced-10* mutant worms. **a** L4 worms expressing the reporter GFP::LGG-1 (*adIs2122* [P*lgg-1*::GFP::*lgg-1; rol-6(su1006)*] in hypodermal seam cells, without (left panel, wild type) and with (right panel) the *ced-10(n3246)* mutation*.* GFP::LGG-1 puncta are labeled with an filled arrow. Magnification bar is 5 μm. **b** The bar graph indicates the number of GFP::LGG-1 foci per cell at the indicated genotypes. These results are mean ± SEM of three independent experiments performed in triplicate. Statistics is Student’s *t* test. ***P ≤ 0.001. **c**, **d** Worms expressing the autophagy reporter SQST-1::GFP (*bpIs151*[P*sqst-1*::*sqst-1*::GFP]) were crossed with *ced-10(3246)* animals and the GFP fluorescence intensity (FI) was analyzed under a fluorescence (**c**) or a confocal (**d**) microscope. **d** (upper panel) L4 animals expressing the array SQST-1::GFP without induction of the GFP reporter in normal conditions. **d** (bottom panel) The *ced-10*(*n3246*) mutation increased GFP intensity and aggregation. Magnification bar is 20 μm. **e** Normalized fluorescence intensity (FI) observed in SQST-1::GFP animals without and with the *ced-10*(*n3246*) mutation. Thirty animals were analyzed per genotype. Data are represented as the mean ± SEM and were obtained by comparing wild type animals (without the *ced-10(n3246)* mutation) with *ced-10* mutated animals. Statistics, ***P < 0.001, Student’s *t* test

Autophagy is considered one of the main pathways involved in α-SYN clearance [19, 46]. Given the role of *RAC1/ced-10* detected in α-SYN levels and in α-SYN-induced DAn cell death, we further sought to determine the participation of *ced-10* in the modulation of autophagy in *C elegans*. To this end, we first crossed *ced-10* mutant animals with those carrying the array *adIs2122 [Plgg-1*::*gfp*::*lgg-1]*. The gene *lgg-1* encodes a ubiquitin-like protein belonging to the Atg8/LC3 protein family, and the respective GFP::LGG-1 translational fusion thus allows to monitor autophagosome formation via fluorescence microscopy [47]. To explore the role of *ced-10* in autophagy, we manually counted the number of GFP::LGG-1 puncta present in the seam cells [48]. At the L3 stage, the number of puncta present in the seam cells were increased in animals harboring the *ced-10* mutation, in comparison to animals without the mutation, in where the GFP::LGG-1 pattern is mainly diffuse (Fig. 4a, b). An increase in the number of GFP::LGG-1 puncta may result from either elevated or impaired autophagic flux [48, 49]. Therefore, we investigated the involvement of *RAC1/ced-10* in autophagic pathways by analyzing the impact of the *ced-10* mutation in the autophagy-associated reporter strain *bpIs51* [P*sqst-1*::*sqst-1*::*gfp + unc-76*(*+*)]. The *C. elegans* SeQueSTosome-related protein, SQST-1, exhibits sequence similarity to mammalian SQSTM1/p62 and is degraded by autophagy [48, 50]. As such, autophagy impairment is often associated with SQST-1::GFP accumulation [48–50]. Similarly to the results obtained with the GFP::LGG-1 reporter, *ced-10* mutant animals displayed increased SQST-1::GFP internal density (Fig. 4c–e). Whereas SQST-1::GFP staining was barely detected in wild type animals (Fig. 4c, d upper panels, and e), *ced-10* worms displayed SQST-1::GFP accumulation (Fig. 4c, d bottom panels, and e). Cumulatively, these results suggest a role of *RAC1*/*ced-10* in the regulation of autophagy.

### Human RAC1 Expression Reduces α-SYN Accumulation and Amyloidogenic Aggregation in a Neuroblastoma Cell Line

**Fig. 5:**
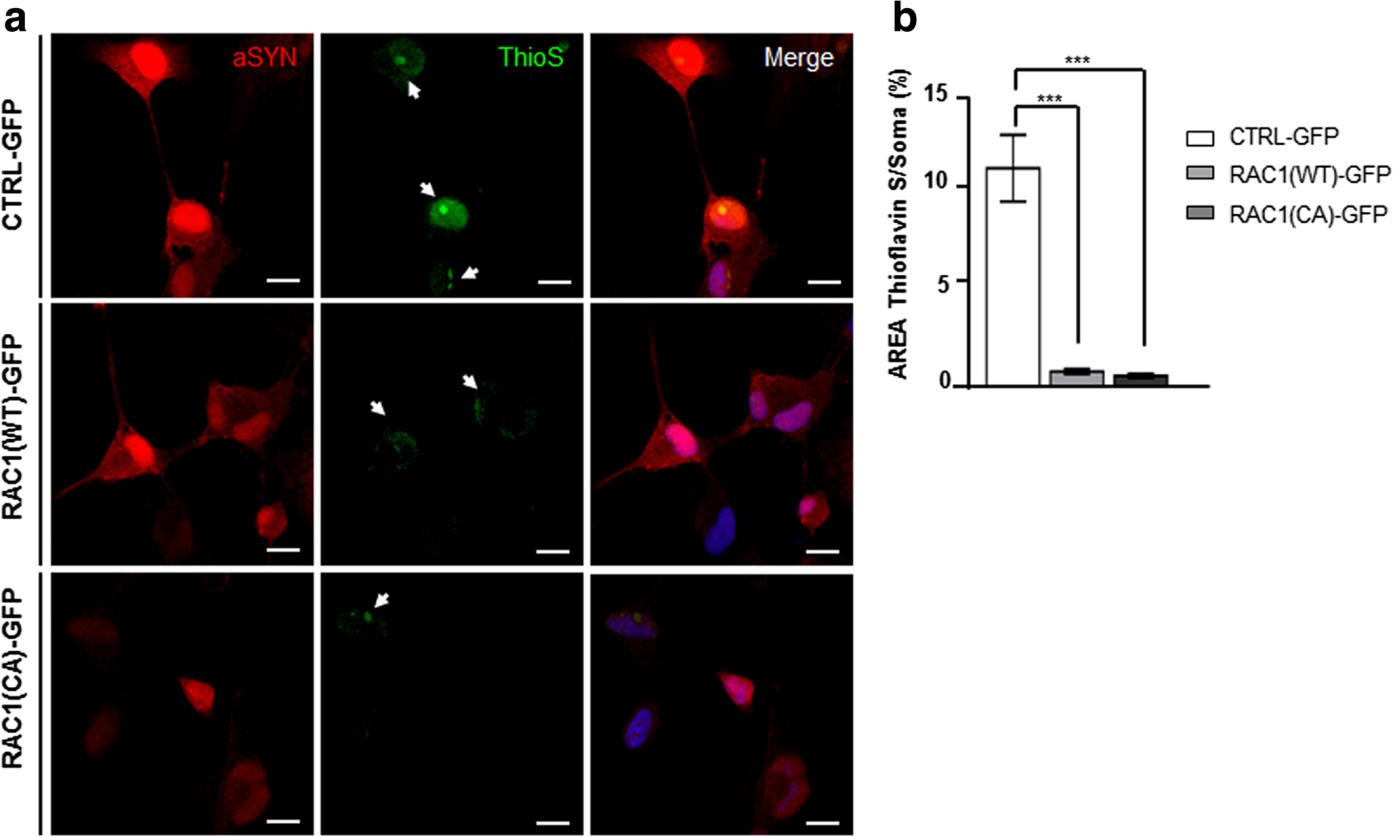
Rac1 activity decreases α-SYN accumulation and aggregation in the neuroblastoma cell line BE(2)-M17. **a** Representative confocal images of α-SYN over-expressing cells induced with 10 μM retinoic acid (RA) and treated with 10 mM sodium butyrate (SB) for 36 h. Cells were transduced with Control-GFP (upper row), RAC1 (WT)-GFP (middle row) and RAC1 (CA)-GFP (bottom row), and co-stained for Thioflavin S (green) and α-SYN (red). Arrows indicate Thioflavin S positive aggregates with amyloidal structure. **b** Bar graph showing the quantitative analyses of the neuronal soma area (in percentage %) covered by Thioflavin S positive stain in individual cells transduced with (WT)- or (CA) RAC1 or with the corresponding control. *N* = 14 (EV), *N* = 25 (WT), and *N* = 24 (CA), from at least three independent experiments. Data are presented as mean ± SEM. Statistics, ***P < 0.001, one-way ANOVA with Bartlett’s test correction followed by post hoc Tukey test. Scale bars represent 10 μm

We further explored the effect of *RAC1* on α-SYN accumulation using a stable BE(2)-M17 neuroblastoma cell line over-expressing wild type α-SYN. As previously reported [51], differentiation with retinoic acid (RA) and treatment with the histone deacetylases inhibitor sodium butyrate (SB) increased α-SYN expression by twofold and induced the accumulation of small α-SYN cytoplasmic aggregates (Online Resource 5a–b). Differentiated cells treated with SB were transduced with a lentiviral vector (LV) expressing either RAC1 wild type (WT)-GFP (in which RAC1-GTP and RAC1-GDP coexist) or RAC1 constitutively active (CA)-GFP (only expressing RAC1-GTP) and analyzed 4 days post-transduction, using the empty vector (Control-GFP) as a control. Infection with both, RAC1 (WT) and RAC 1(CA) decreased α-SYN expression level (Online Resource 5b) and aggregation, as shown by Thioflavin S (ThyoS) dye (Fig. 5), which specifically stains cross-beta sheet fibrils, such those forming amyloid aggregates [52]. The area covered by ThyoS -positive α-SYN aggregates per cell was decreased by 90% in RAC1(WT) and RAC1(CA) infected cultures (Fig. 5) thus suggesting a role of RAC1 in either the formation or clearance of toxic α-SYN species and corroborating the data obtained by western blot in the nematode.

### High RAC1 Activity Reduces α-SYN Levels and Increases Neurite Arborization in PD Patient-Specific Midbrain iPSC-Derived DAn

Lastly, to connect the nematode data with the human PD, we differentiated DAn upon cell reprogramming of skin fibroblasts into induced pluripotent stem cells (iPSC) from PD patients carrying the G2019S (G/S) mutation in the *LRRK2* gene. While preserving the patient genetic background, this model exhibits some characteristic features and cellular phenotypes of PD including reduced axonal outgrowth, α-SYN accumulation, α-SYN-induced DAn cell death, and impaired autophagy [20]. Therefore, it represents a suitable tool to contextualize and compare the nematode encountered data.

**Fig. 6:**
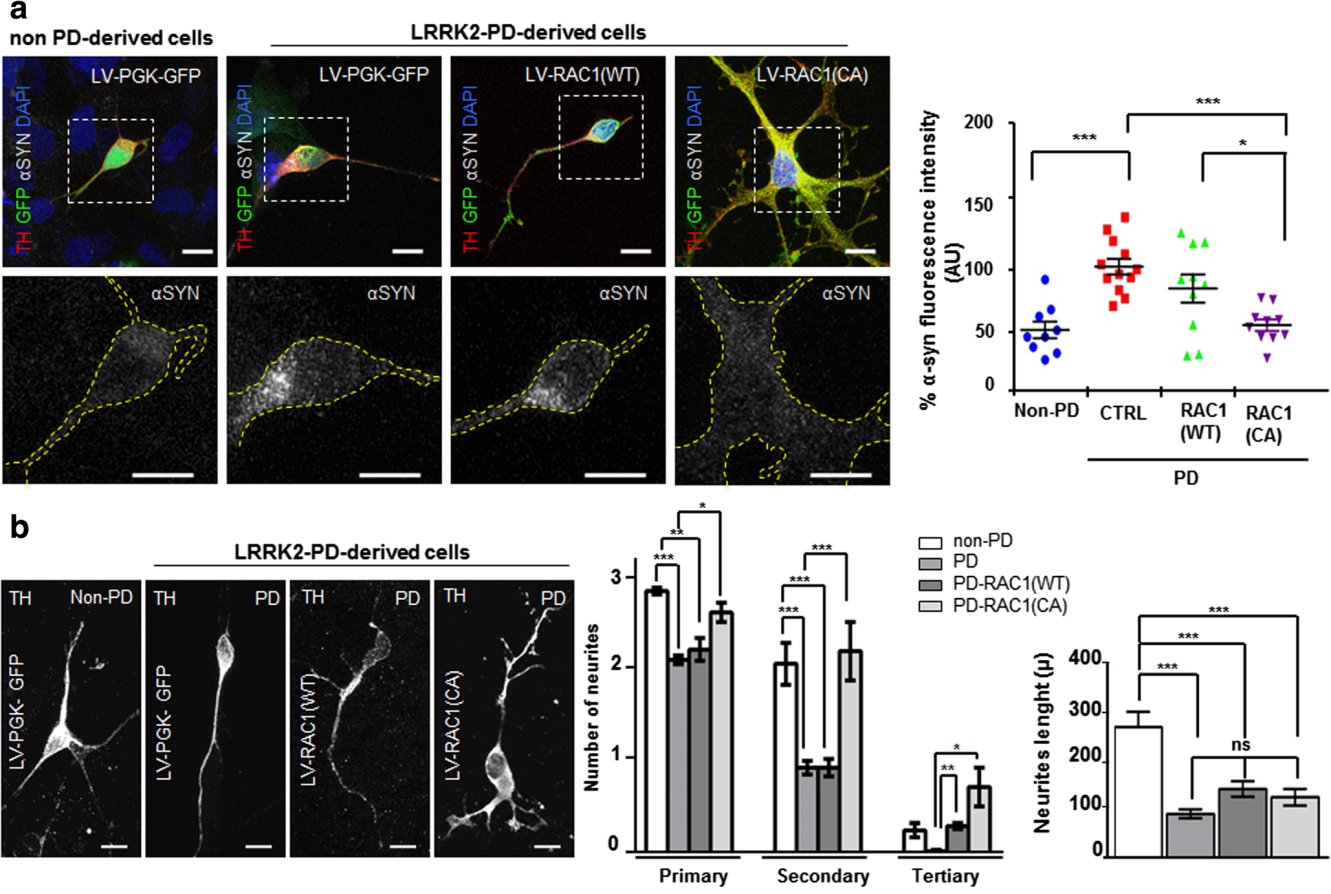
Rac1 activity rescues α-SYN accumulation and neurite degeneration in early LRRK2-PD-derived DAcells. **a** First row shows confocal images of non-PD (first panel) and LRRK2-PD-iPSC-derived DAn (second, third, and fourth panels) at 30 days of differentiation transduced with Control-GFP (first and second panels), RAC1 (WT)-GFP (third panel) and RAC1 (CA)-GFP (fourth panel), and co-stained for GFP (green), Tyrosine hydroxylase (TH) (red) and α-SYN (gray). Nuclei are counterstained with DAPI, shown in blue. Second row shows confocal images representing the expanded pictures of the corresponding above neurons highlighted within the white dashed square, evidencing α-SYN staining. Dot plot shows the quantification of the average (in %) of α-SYN fluorescent intensity in every analyzed neuron positive for TH and GFP. Statistical analysis is the result of comparing α-SYN staining intensity of non-PD with LV-transduced DAn. Data is the average of at least two-independent experiments and are presented as mean ± SEM. Statistics is one-way ANOVA with a Tukey’s post hoc analysis **P < 0.01. **b** Representative confocal micrographs of single DAn derived from non-PD (first panel) and LRRK-2PD patients, transfected with Control-GFP (second panel), RAC1 (WT)-GFP (third panel) or RAC1 (CA)-GFP (fourth panel). Extension bars are 10 μm. (Left graph) This bar graph represents the number of neurites per neuron (primary, secondary and tertiary), according to the indicated transduction, in non-PD and in PD-derived cells. Statistical analysis is the result of comparing neurite number non-PD with LRRK2-PD-derived DA cells transduced with RAC1 (WT)-GFP or RAC1 (CA)-GFP. Data is the average of at least two-independent experiments and are presented as mean ± SEM. Statistics is One-way ANOVA, *P < 0.05 and **P < 0.01. (Right graph) Quantitative analyses of the neurite length (in μm) in DAn derived from non-PD and LRRK2-PD-derived patients. Statistical analysis is the result of comparing neurite number non-PD with LRRK2-PD-derived DA cells transduced with RAC1 (WT)-GFP or RAC1 (CA)-GFP. Data are presented as mean ± SEM. Statistics is one-way ANOVA. ***P < 0.001

At 30 days of differentiation, patient iPSC-derived DAn do not show overt morphological signs of neurodegeneration [20]. However, almost 40% of DAn positive for the DA marker tyrosine hydroxylase (TH) showed detectable amounts of α-SYN [20]. For this reason, and to explore early events affecting PD, we next investigated the contribution of RAC1 in PD by rescuing α-SYN accumulation in DAn. For this purpose, PD-iPSC-derived midbrain DAn (at 30 days of differentiation when α-SYN accumulation is already evident), were transduced with lentivirus (LV) expressing either RAC1 wild type (RAC1 (WT)-GFP or a highly active form of RAC1 (RAC1 (CA-GFP), and LV-GFP as control (Control-GFP) (Online Resource 6a, c and d), and analyzed 7 days after transduction. We found that LRRK2-PD-derived DAn, transduced with Control-GFP showed significant increase in α-SYN content in comparison with non-PD-derived DAn (Fig. 6a, first and second panels, and scatter dot plot) confirming previous results [20]. A 18% decrease in α-SYN accumulation was observed in PD-derived cells infected with RAC1 (WT)-GFP, and it was even stronger (48.15%) in PD-derived cells infected with RAC1 (CA)-GFP (Fig. 6a, third and fourth panels, and scatter dot plot, ***P < 0.001). By analyzing the number and length of neurites, to explore the capacity of Rac1 in rescuing neuronal degeneration (Online Resource 7), we found a decrease in neurite arborization in PD-derived DAn (Fig. 6b), confirming previous reports showing reduction in neurite length/branching and defects of Rac signaling in LRRK2-associated parkinsonism [53]. Importantly, overexpression of RAC1 (CA)-GFP, but not RAC1 (WT)-GFP, was associated with significant increase of neurite arborization (Fig. 6b, fourth panel and left graph), consistent with a role for RAC1 in organizing the actin cytoskeleton [58]. The neurite length was not rescued in any of the conditions tested (Fig. 6b, right graph).

By transcriptome analysis we have previously reported that iPSC-derived DAn from PD patients exhibited a large number of gene expression changes. More specifically, we identified 437 differentially expressed genes (DEGs) in PD vs. controls of which 254 were up-regulated in PD patients and 183 were down-regulated [54]. Here, to gain further insight into the canonical pathways affected by differential gene expression detected at early PD, we performed a biological enrichment analysis at 30 days of differentiation, by using the software and databases of Ingenuity Pathway Analysis (IPA). We found that the signaling by Rho family GTPases pathway was the top-1 statistically most significant canonical pathway in PD patients compared to controls (*P* = 2.56 × 10^−4^) (Online Resource 8). From the total of 245 members comprised in this pathway, 16 DEGs of which 9 genes were up-regulated and 7 down-regulated in PD DAn (Online Resource 9). Interestingly, the top 2 statistically most significant canonical pathway was the related Rho GDI pathway (*P* = 7.91 × 10^−4^). Overall, the results from this unbiased biological enrichment analysis identifies Rho family GTPases as top deregulated canonical pathway in iPSC-derived DAn from PD patients.

### RAC1 Activity Increases the Long-Term Survival of PD-Patient-Derived DAn and Alleviates the Impairment of Autophagy

To assess whether the protective effect of RAC1 in reducing α-SYN levels correlated with increased survival rates over time, neurons were further cultured for 75 days (Online Resource 6b) by co-culturing them over a monolayer of mouse post-natal cortical astrocytes [55], which supported viable cultures of DAn for up to 75 days [20].

**Fig. 7:**
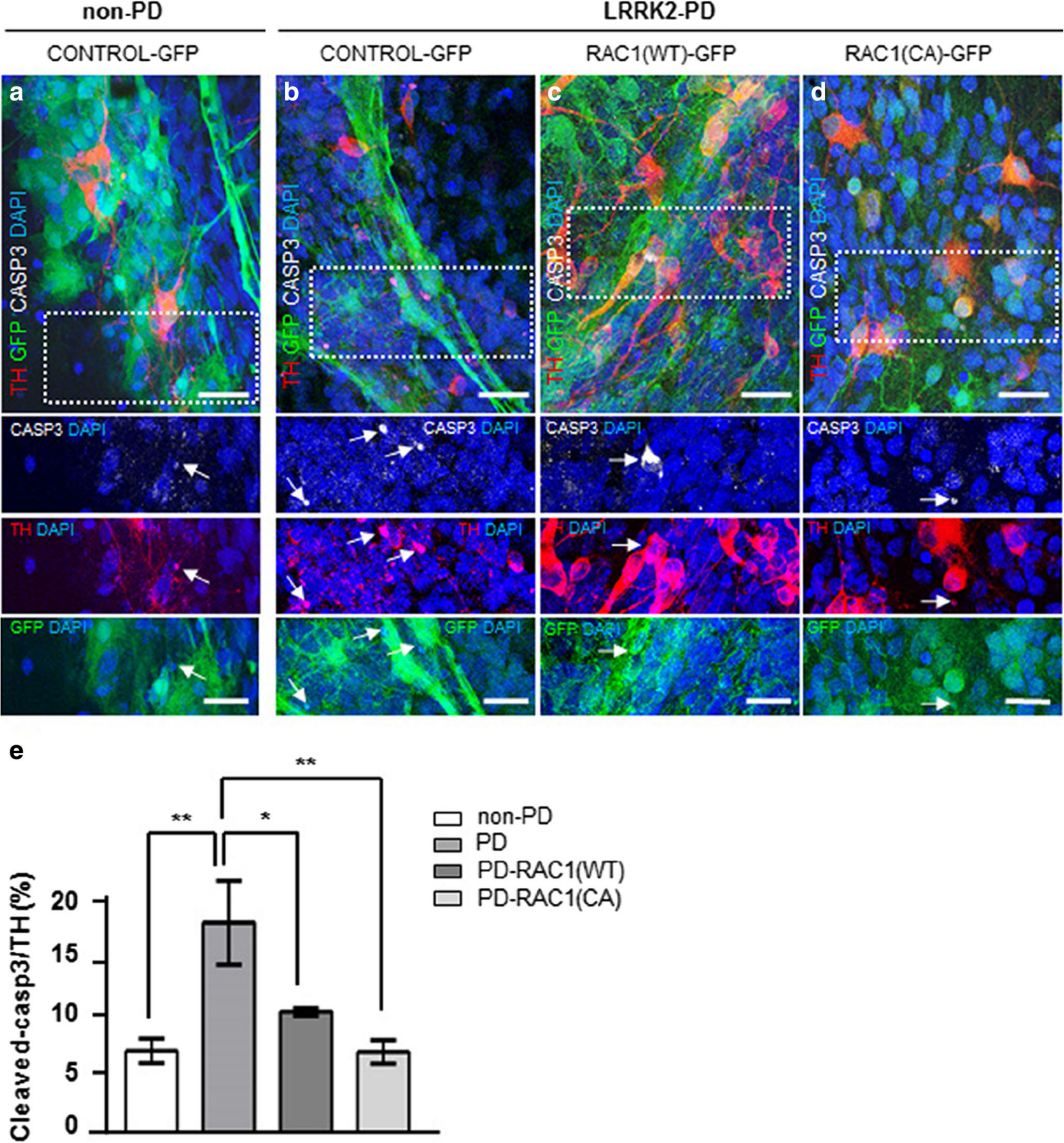

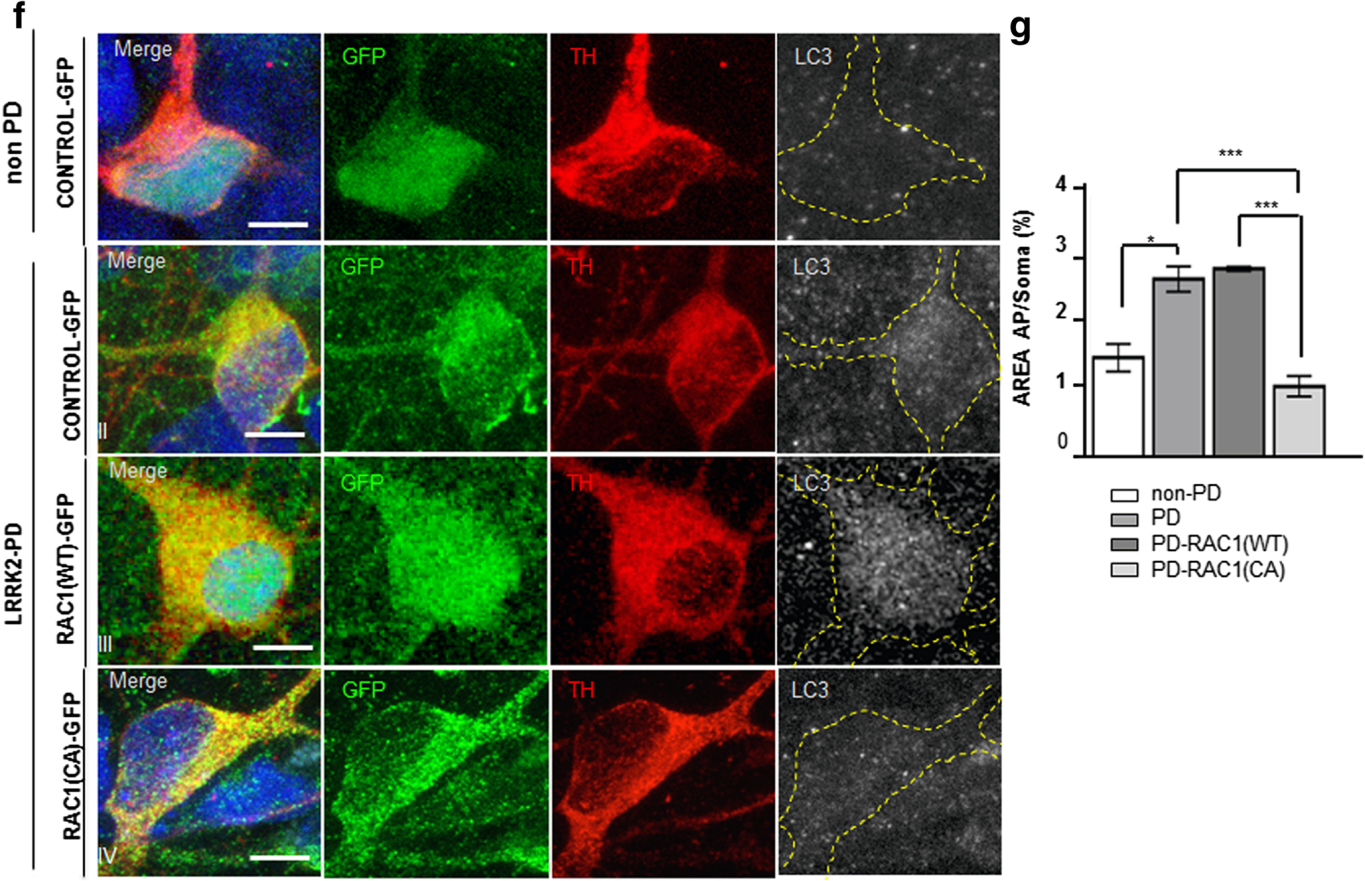
RAC1 activity increases the long-term survival of PD-derived DAn and alleviates autophagy impairment. (A–D) Confocal images of non- PD (A) and PD-iPSC-derived DA cultures transduced with Control-GFP (B), RAC1 (WT)-GFP (C), and RAC1 (CA)-GFP (D), stained for GFP (green), TH (red) and cleaved Caspase-3 (white). A representative area was highlighted in dashed lines and expanded in below panels. The corresponding lower insets show higher magnification images of each separate channel, cleaved caspase-3, TH and RAC1-GFP respectively. TH+ neurons, stained with Caspase-3 and transduced with RAC1-GFP constructs are labeled with white arrow tips. Caspase-3 posoNuclei are counterstained with DAPI, shown in blue. PD-derived cells transduced with Control-GFP (second panel) showed increased numbers of triple positive TH/GFP/Caspase 3 staining in TH+ neurons in comparison to the other conditions tested, where cells were non-PD (first panel left) or transduced with RAC1 (WT)-GFP and RAC1 (CA)-GFP (third and fourth panels respectively). Extension bar from A–D is 20 and 10 μm for top and bottom panels respectively. **e** Bar graph showing the quantification of the number of TH/GFP double-positive neurons presenting cleaved Caspase-3 staining. Data is the average of at least two-independent experiments and are presented as mean ± SEM. Statistics, *P < 0.05, **P < 0.01, and ***P < 0.001, Two-way ANOVA, Tukey post hoc test. **f** Confocal images of non-PD (left panel) and PD-iPSC-derived DA cultures transduced with Control-GFP (second row), RAC1 (WT)-GFP (third row), and RAC1 (CA)-GFP (fourth row), stained for GFP (green), TH (red) and LC3 (gray). Non-PD cells (first row) and PD cells transduced with RAC1 (CA)-GFP (fourth row) showed similar amount of LC3-II positive vesicles in TH+/GFP+ neurons. Nuclei are counterstained with DAPI, shown in blue. Extension bar is 5 μm. **g** Bar graph showing the quantification of the neuronal soma area (in percentage %) covered by LC3-II positive vesicles in at least 15 DA neurons. Data is the average of at least two-independent experiments and are presented as mean ± SEM. Statistics, *P < 0.05, Two-way ANOVA, post hoc Tukey test

After this time span, differentiated cultures from genetic LRRK2-PD- patient derived DAn showed higher numbers of apoptotic DAn when compared to those derived from healthy subjects [20] and Fig. 7(a, b, and e). Overexpression of both RAC1 (CA)-GFP and RAC1 (WT)-GFP prevented cell death by reducing the amount of DAn positively stained for cleaved caspase-3 to the levels of the non-PD-patient-derived DA cells (Fig. 7(c–d and e)). We and others have described that LRRK2 G2019S mutation has negative effects in the autophagic flux by seemingly impairing autophagosome-lysosome fusion [20]. In this specific case, RAC1 CA (RAC1-(CA) but not RAC1 wild type (RAC1-(WT) displayed autophagosome vesicle numbers similar to those of the non-PD-patient-derived neurons (Fig. 7). Therefore, these results suggest a mechanism by which a better performance of the autophagic clearance promoted by RAC1 alleviates the accumulation of aggregation-prone proteins, such as α-SYN, thus contributing to increase the survival of DAn.

In conclusion, these results obtained in DAn derived from LRRK2-associated PD patients are in line with findings in the nematode models of PD, where RAC1 activity is directly involved with DAn survival in the presence of α-SYN, α-SYN inclusions formation and autophagic mechanisms.

## Discussion

Here, we demonstrate in *C elegans* and in human-derived PD cells that *RAC1/ced-10* participates in the main pathogenic manifestations of PD such as DAn death, α-SYN accumulation and impaired autophagy. Besides, the results obtained in the nematode, suggest a role of *ced-10* modulating DA behavior in the presence of α-SYN. Furthermore, *RAC1* function is associated with the considered toxic α-SYN species. Overall, in this manuscript we propose *RAC1/ced-10* as a potential therapeutic target for the treatment of PD-related disorders.

### *RAC1/ced-10* and DAn Death

Previous research has shown that Rac GTPases play an essential neuroprotective and pro-survival role in neuronal models and diseases [17, 56–58]. Indeed, our in depth analysis of the RAC1 signaling pathway arose from the transcriptomic data in human iPSC-derived DAn from PD patients, showing altered Rho signaling as top deregulated pathway, points to this same direction (**Online Resources** **8****–****9**). However, Rac GTPases modulation in different cell types is much more complex. Loss of RAC GTPase activity may contribute to the death of DAn while increased Rac-GTP activity in microglia may contribute to the formation of toxic ROS [59]. Hence, this complicated RAC1 modulation depending upon the tissue and the ROS state, might explain the behavioral differences observed between whole *ced-10* mutant animals and *RAC1/ced-10* specifically depleted in DAn in the presence of α-SYN (Fig. 1). Interestingly, a cell non-autonomous function for hypodermal *RAC1/ced-10* in the maintenance of axonal survival has been recently proposed in *C. elegans* [60]. Consequently, the influence of RAC1 activity in the neighboring tissues cannot be obviated.

There exist positive correlation between neuronal apoptosis and decreased RAC1 GTPase activity [17]. Very different cellular models, such as human lymphoma cells or primary cerebellar granule neurons, suggest the inhibition of caspase-induced apoptosis by RAC1, whereby AKT-dependent pro-survival pathways and the consequent Bcl-2-associated death protein (BAD) phosphorylation were downstream and activated by RAC1 [59, 61].

The activation of the AKT by RAC1, also participates in the cytoskeleton reorganization and cellular growth [62, 63] and a failure to maintain the integrity of DAn after they are formed could cause DAn death [64]. Accordingly, RAC1-modulated processes involved in the maintenance of cell integrity, might be crucial for cell survival.

LRRK2-PD-patient-derived DAn show increased neurite numbers after being transduced with RAC1 (Fig. 6), thus expanding the role of this GTPase in the maintenance and in the generation of new neurites [13], and thus contributing accordingly to DAn survival (Fig. 7). Therefore, our results are in accordance with LRRK2 inducing neurite retraction through diminished RAC1 GTPase activity [16]. Surprisingly, neurite length was not rescued with any of the infected RAC1 constructs in the present manuscript. Differences between results can be explained based on the different cellular models used in both laboratories, since our results are provided directly from PD-derived cells, whereas the neuroblastoma cell line SHSY5Y is the model used by Chan et al. [16]. In addition, the existing actin-microtubule cross talk in the process of neurite outgrowth and elongation has to be considered [65, 66]. Microtubules are the main cytoskeletal components of neurites [66] and decreased stability of microtubules is a common feature of neurodegenerative diseases [67]. Interestingly, LRRK2-PD variants are characterized by defects in microtubule associated processes [68] and LRRK2 regulates microtubule stability [69].However, extension and navigation of neurites are normally driven by actin-rich growth cones and inhibition of microtubules dynamics does not stop neurite outgrowth [66]. Thus, our results are consistent with cellular dysfunction in PD, with RAC1 modulating actin-associated mechanisms better than in microtubule linked processes.

### *RAC1/ced-10* and α-SYN Accumulation

One of the main factors linked with DAn death in PD progression is α-SYN accumulation. α-SYN overexpression in model systems, and its concomitant aggregation and deposition precede neuronal cell death. In the case of DAn, its degeneration is influenced by intracellular and extracellular α-SYN accumulation, mainly in its oligomeric form [70]*.* Interestingly, extracellular oligomeric α-SYN impairs RAC1 activity in neuroblastoma cells [70]. Considering the increased intracellular α-SYN aggregates together with increased oligomeric α-SYN species observed in *ced-10* mutant animals, we hypothesize that altered *RAC1*/*ced-10* function might accelerate α-SYN accumulation and the formation of α-SYN oligomers which might bind concurrently *RAC1/ced-10* [70] thus increasing the severity of *RAC1/ced-10* altered function. Moreover, and considering the modulation of the actin cytoskeletal dynamics by α-SYN [71], a synergistic regulation between RAC1 and α-SYN cannot be excluded.

Additional experiments, where the amount of α-SYN could be tightly controlled, will provide some clues about the relevance of RAC1/α-SYN interaction in the progress of PD.

α-SYN accumulation in PD patients is associated with failure of the two major protein breakdown pathways, the ubiquitin proteasome system (UPS) and autophagy [72–74], which, in cooperation, reduce the misfolded protein burden [75]. Stably increased levels of α-SYN can lead to impaired proteasome function [76] and *ced-10* is proteasome regulated in the phagocytosis of dead cells [14]. Hence, we hypothesize that increased α-SYN in a *ced-10* mutant background might reinforce the severity of the *ced-10* mutation in the degradation of α-SYN, due to the interaction of α-SYN with the proteasomal machinery.

Overexpression of α-SYN results in the inhibition of autophagy [77]. Here we suggest *RAC1/ced-10* being necessary for autophagy to occur (Figs. 3 and 7). Accordingly, α-SYN accumulation, due to *ced-10* impairment, might increase the severity of *ced-10* mutation. Therefore we add RAC1 to the already proposed feedback loop between proteasome activity and autophagy [1] and we propose that a tight regulation of RAC1 function is required to avoid excess of α-SYN accumulation and the concomitant cell death.

Impaired autophagy associated with LRRK2 mutations are already reported [78, 79], with the G2019S mutation showing less autophagic activity [80]. In the context of LRRK-2 induced phenotypes, we propose RAC1-LRRK2 interaction as relevant factor favoring autophagy to occur, and helping in the clearance of α-SYN aggregates, thus warranting the proper neurite growth and maintenance.

Future scientific research is needed to unravel the mechanisms associated with PD-related disorders for finding efficient therapies. In light of our results the pharmacological modulation of RAC1 and RAC1-derived signaling pathways could be of therapeutic value.

## Experimental Procedures

### Worm Experiments

#### *C. elegans* Strains

Nematodes were maintained using standard procedures [81]. We obtained the strains CB1112 *cat-2(e1112),* NL5901, *unc-119(ed3) III;* [*pkIs2386* (P*unc-54*::*α-SYN*::*YFP; unc-119(+)*)]; DA2123 *adIs2122* [P*lgg-1*::*lgg-1*::*GFP*; *rol-6(su1006)]* and HZ589, *bpIs151* [P*sqst-1::sqst-1*::GFP*; unc-76(+)*] *IV; him-5(e1490) V* from the *Caenorhabditis elegans* Genetic Center (CGC). The strain BR3579, *ced-10(n3246)* was a generous gift from Dr. Ralf Baumeister (Albert-Ludwing University, Freiburg/Breisgau, Germany).

The strain BR3579 was crossed with NL5901 animals to generate the strain EDC101, *unc-119(ed3) III; pkIs2386* [P*unc-54*::*α-SYN*::YFP*; unc-119(+)*]*; ced-10(n3246).*

The following strains is used to analyze the DAn degeneration as reported in Harrington et al., 2010 [36]: P*dat-*_*1*_::α-SYN +P*dat-1*::GFP from the main text is named UA196*,* with the genotype*: sid-1(pk3321); baIn33* [P*dat-1*::*sid-1*; P*myo-2*::*mCherry*]*; baIn11* [P*dat-1*::α-SYN*; Pdat-1*::GFP]*.*

For autophagy experiments, males from the DA2123 strain [P*lgg-1*::*lgg-1*::GFP*; rol-6(su1006)]* were crossed with *ced-10(n3246)* hermaphrodites. Males a from the HZ589 *bpIs151* [P*sqst-1*::*sqst-1*::GFP*; unc-76*(*+*) *IV*]*; him-5*(*e1490*) *V*] strain, expressing SQST-1::GFP crossed with *ced-10* (*n3246*) hermaphrodites.

N2 (Bristol) was used as the *C. elegans* wild-type (wt) strain. Hermaphrodites were used throughout of the study.

#### RNAi Feeding

For feeding RNAi bacteria, egg lay from gravid adults were directly transferred to NGM plates containing 1 mM of isopropyl β-D-1-thiogalactopyranoside/ IPTG (referred to as RNAi plates) seeded with 20X concentrated bacteria containing 50 μg/ml ampicillin, carrying desired plasmid for RNAi of a specific gene (*ced-10* or *cat-1*, depending upon the experiment) or bacteria carrying empty (EV) pL4440 as control and allowed to grow on plates for approximately 50 h to reach the L4 stage and then another 16 h for the conduction of actual experiment.

Note: Bacterial clones for RNAi feeding protocol were obtained from the Ahringer library (Source Bioscience, Nottingham, UK) [82] and were then streaked on LB-Tetracycline-Ampicillin plate which was then incubated at 37 °C. Individual colonies from this freshly streaked plate were grown for 10–12 h constantly shaking at 37 °C in LB medium containing 50 μg/ml ampicillin.

#### Blinding of Experiments and Replicates

All behavioral studies were completed such that the experimenter was blind to the genotype of the worms. Strains were given letter codes by another member of the laboratory and the code was not broken until all of the replicates for a particular assay were completed. For all assays, we completed a minimum of three biological replicates per strain.

#### Behavioral Experiments

##### Locomotor Rate Assay

Locomotor rate assay was performed as described in [33]. Briefly, assay plates were prepared by spreading the *E. coli* strain OP50-1 in a ring on NGM agar [81] in 5-cm petri plates. Assay plates were always freshly spread with bacteria, incubated overnight at 37 °C, and allowed to cool to room temperature before use. Plates for measuring locomotor rate in the absence of bacteria were also incubated at 37 °C. Only synchronized young adult hermaphrodites (16 h after the late L4 larval stage) were tested. For well-fed animals, locomotor rate was measured by removing 5 animals from plates with ample bacteria, washing the animals twice in S basal buffer [81], and transferring them to an assay plate in a drop of buffer using a capillary pipette. The drop of buffer used to transfer the animals was absorbed with a Kimwipe. Five minutes after transfer, the number of body bends in 20 s intervals was sequentially counted for each of the five animals on the assay plate and then repeated the same thing for next set of animals in a different assay plate.

##### Ethanol Avoidance Assay

Ethanol avoidance assay was performed as described [34] (Cooper et al. 2015). Synchronized young adult hermaphrodites (16 h after the late L4 larval stage) were transferred to assay plates, which are divided into four quadrants: two quadrants seeded with 50 μl of 100% ethanol and the others without. Worms are placed in the center of the assay plate and allowed to move for 30 min at which point the entire plate is imaged and the worms are scored for their quadrant of preference. Ethanol avoidance is calculated as [(number of worms in control quadrants) − (number of worms in ethanol quadrants)]/total number of worms.

##### Generation of the Rescue Construct P*ced-10*:: CFP::*ced-10*

P*ced-10*::CFP::*ced-10* plasmids were co-injected into worm strain EDC101 and UA196 to generate UA281, *baEx167* [P*ced-10*::CFP::*ced-10, rol-6(su1006)*]; [P*unc-54*::*α-*SYN::YFP*;ced-10(n3246)*] and UA282, *baEx167* [P*ced-10*::CFP::*ced-10; rol-6(su1006)*]*; sid-1(pk3321)*; *baIn33* [P*dat-1*::*sid-1,* P*myo-2*::*mCherry*]; *baIn11*[P*dat-1*::*α-*SYN; P*dat-1*::GFP]). Hermaphrodites were used throughout of the study.

##### Site-Directed Mutagenesis

The construct P*ced-10*::GFP::*ced-10* was provided by Erik Lundquist (University of Kansas, Lawrence, KS, USA) as a gift. TagMaster Site-directed mutagenesis (GM Biosciences, Rockville, USA) was used to create mutations (Y66W, Y145F, and M153T) in GFP sequence for changing fluorescence marker as CFP. The product plasmid P*ced-10*::CFP::*ced-10* was sequenced (Eurofins Genomics, Huntsville, AL, USA) to confirm the presence of the desired mutations.

##### *C. elegans* Neurodegeneration Assays

Worms were analyzed for DA neurodegeneration as described previously [38]. Briefly, 10 L1-stage worms (neuron-specific RNAi worm strain with α-Syn UA196 were transferred to the plates (empty vector (EV) and *ced-10* RNAi) and grown at 20 °C until adulthood. Adult worms were then transferred to corresponding freshly made RNAi plates and allowed to lay eggs for 6 h to synchronize. α-SYN-induced DA neurodegeneration was scored at the indicated days after hatching (L4 + 7; L4 + 3; L4 + 1). To investigate the effect of CED-10 overexpression on α-SYN –induced DA degeneration, the strain UA282 was analyzed at L4 + 7 days of aging. Worms were considered normal when all six anterior DA neurons (four CEP (cephalic) and two ADE (anterior deirid)) were present without any visible signs of degeneration. If a worm displayed degeneration in at least one of the six neurons, it was scored as exhibiting degeneration. In total, at least 50 adult worms were analyzed for each independent transgenic line or RNAi treatment.

##### Aggregate Quantification

The quantification of aggregates was performed as previously described [83]. Briefly, NL5901animals without and with *ced-10(n3246)* mutation, together with the EDC101, were age-synchronized by bleaching with NaOCl and left overnight to hatch. L1 animals were transferred onto individual NGM plates seeded with *Escherichia coli*. Aggregates were counted for each animal staged at L4 + 5 days. Images were captured using a Leica SP5 confocal microscope with a ×40 oil immersion lens (HCX PL APO CS). The number of α-syn aggregates was determined in the mid body of each animal, taking the vulva position (V) as a reference. Aggregates were defined as discrete, bright structures, with boundaries distinguishable from surrounding fluorescence. Measurements on inclusions were performed using ImageJ software taking into consideration the area dimensions.

##### Thrashing Assays

At L4 + 5 days, animals from the strains N2 wild type, NL5901 [*unc-119(ed3) III; pkIs2386* [P*unc-54*::*α-*SYN::YFP*; unc-119(+)*] and EDC01 *unc-119(ed3) III; pkIs2386* [P*unc-54*::α-SYN::YFP*; unc-119(+)*]; *ced-10 (n3246)* were placed in a drop of M9 buffer and allowed to recover for 120 s (to avoid observing behavior associated with stress) after which the number of body bends was counted for 1 min. Movies of swimming worms were recorded using a Leica MZFFLIII stereomicroscope at nominal magnification of 30X and the Hamamatsu ORCA-Flash 4.0LT camera at 17 frames per second (17 fps) for 1 min. Bends per minute were obtained with the Worm Tracker pluggin (wrMTrck), from the ImageJ software [84]. Thirty animals were counted in each experiment unless stated otherwise. Experiments were carried out in triplicate. Statistical analysis was performed using Graphpad Prism version 6.00 for Windows, GraphPad Software, La Jolla, CA, USA).

##### Detergent Fractionation

α-SYN oligomeric species were isolated by detergent fractionation as described (Kuwahara et al. 2012) with slight modifications. Briefly, worms were washed three times with M9 buffer, collected as a 100 μl pellet, and the pellet was snap-frozen in liquid nitrogen. The pellets were homogenized in 1 mL of 50 mM Tris-HCl buffer at pH 7.5 with complete protease inhibitor mixture (Roche Applied Science) by brief sonication. Sonicates were centrifuged two times at 1000×*g* for 5 min to remove debris of worm tissue. The supernatant was then ultracentrifuged at 350,000×*g* for 15 min, and the supernatant was collected as a Tris-HCl soluble fraction. The resulting pellet was subsequently extracted by sonication in 500 μl of Triton X-100 (Tris-HCl buffer with 1% TritonX-100), 200 μl of Sarkosyl (Tris-HCl with 1% Sarkosyl), and 100 μl of SDS (TrisHCl with SDS sample buffer containing 2% SDS) followed by centrifugation at 350,000×*g* for 15 min. The Tris-HCl fraction containing 20 μg of total proteins, along with equal volumes of Triton X-100, Sarkosyl, and SDS fractions to the Tris-HCl fraction, were loaded onto the acrylamide gel and separated by SDS-PAGE.

##### Immunoblotting

Twenty microliters of protein per strain were loaded from each sample and fraction on Novex NuPAGE 4–12% Bis-Tris Gels (Thermo Fisher) an run in MES SDS buffer at 200 V for 40 min. Resolved proteins were transferred onto 0.45 μm nitrocellulose membranes (Amersham) at 200 mA for 1 h. Blocking was done in 5% milk powder in PBS for 1 h, followed by overnight incubation at 4 °C with a mouse monoclonal antibody against α-synuclein (1:1000, BD Biosciences, #610787) diluted in 2% bovine serum albumin (BSA)/PBS. Incubation with a donkey anti-mouse (1:5000, Amersham) secondary antibody diluted in 1% milk powder/PBS was performed for 1 h at room temperature.

Membranes were developed using Supersignal West Femto (Thermo Scientific) in a ImageQuant RT ECL Imager (GE Healthcare).

##### Autophagy Measurements

For measuring the involvement of *ced-10(n3246)* mutation in autophagy, we followed specific *C. elegans* protocols [48, 85]. LGG-1::GFP foci were analyzed in the hypodermal seam cells at L3 stage of development. Images were not considered for quantification where the hypodermis was not clear. In total, at least 40 total regions were quantified from 30 different worm samples per genotype.

To confirm the involvement of *ced-10* in impairing autophagy, we followed the protocol described in [86]. Pictures of L1 animals (whole animal) and L4 animals (whole intestine), were analyzed by using a 63× objective. The GFP internal intensity (FI), corresponding to SQST-GFP foci, was quantified at L4 stage of development. The presence of the *ced-10* mutation was checked by PCR as described [14]. The presence of the GFP reporter was double checked by PCR using the *gfp* primers, and under the fluorescence microscope.

##### Microscopy and Imaging

For neurodegeneration assays and aggregates quantification animals were mounted with a coverslip on a 4% agarose pad in M9, containing 10 mM of sodium azide. Confocal microscopy was performed using a Leica TCS-SP5 confocal spectral microscope (Leica, Barcelona, Spain) and analyzed using ImageJ software (NIH, ver. 1.43, Schindelin, 2015). Animals were examined at 100× magnification to examine α-SYN induced DA cell death and at 40X to examine α-SYN apparent aggregates.

For autophagy measurements, animals were placed in a 5 μL drop of 10 mM solution of levamisole (Sigma-Aldrich, Madrid, Spain). For each independent experiment, between 20 and 30 7-day-old worms of each treatment were examined under a Nikon EclipseTE2000-E epifluorescence microscope equipped with a monochrome camera (Hamamatsu ORCA-ER) coupled to the Metamorph software (Molecular Devices Corp., Sunnyvale, CA). The system acquires a series of frames at specific Z-axis position (focal plane) using a Z-axis motor device.

### Cell Culture Experiments in BE(2)-M17 Neuroblastoma Cell Line

#### Cell Culture

Human neuroblastoma cell line BE(2)-M17 over-expressing wild type α-SYN were maintained at 37 °C and 5% CO_2_ in Optimem medium (Gibco) (Thermo-Fisher Scientific, Madrid, Spain) supplemented with 10% fetal bovine serum (Gibco) (Thermo-Fisher Scientific, Madrid, Spain), 500 μg/ml G418 (Geneticin) (Sigma-Aldrich, Madrid, Spain) and Penicillin/Streptomycin (Sigma-Aldrich, Madrid, Spain). For immunofluorescence, 2 × 10^4^ cells/well were seeded in 24-well plates on coverslips coated with poly-D-lysine. α-SYN aggregation was induced as previously reported ([51] by differentiation with 10 μM retinoic acid (Sigma-Aldrich, Madrid, Spain) for 10 days, followed by treatment with the histone deacetylases inhibitor sodium butyrate (SB) (Sigma-Aldrich, Madrid, Spain) at a concentration of 10 mM for 36 h. To analyze the effect of RAC1 on α-SYN aggregation, cells were transduced 24 h before SB treatment with either the lentiviral empty vector or with the wild type or the constitutively active isoforms of RAC1 at an estimated multiplicity of infection (MOI) of 5.

#### Immunofluorescence

BE(2)-M17 cells were fixed in 4% paraformaldehyde (PFA) (Sigma-Aldrich, Madrid, Spain) in phosphate saline buffer (PBS) for 30 min at 4 °C and permeabilized with Tris-buffered saline (TBS) containing 0.5% Triton X-100 (Sigma-Aldrich, Madrid, Spain) for 5 min. Cells were then blocked with 3% normal goat serum (NGS) (Vector Laboratories, Palex Medical, Sant Cugat del Vallès, Spain) in TBS for 1 h and subsequently incubated with the corresponding primary antibody in 1%NGS/TBS overnight at 4 °C. The following primary antibodies were used: mouse anti-α-SYN (610,787; 1:500) (BD Biosciences, Madrid, Spain), rabbit anti-α-SYN (2642; 1:500) (Cell Signaling, Leiden, The Netherlands). Incubation with goat secondary antibodies conjugated with Alexa488 (anti-mouse, A11001; or Alexa594 (anti-mouse, A11005; anti-rabbit, A11012) (Thermo-Fisher Scientific, Madrid, Spain) was done in 1% NGS/TBS for 1 h at RT. Between each incubation period cells were rinsed three times in TBS.

#### Thioflavin S Staining

After incubation with the antibodies, coverslips were immersed in 0.005% thioflavin S (Sigma-Aldrich, Madrid, Spain) in PBS for 8 min and then rinsed twice in ethanol 70% and once in PBS. Only after thioflavin S staining, nuclei were counterstained with Hoechst 33342 (Thermo Fisher Scientific, Madrid, Spain, 1:10,000 in PBS) for 10 min.

#### Microscopy and IF Quantification

Cells were cover slipped using Dako Cytomation Fluorescent Mounting Medium (Dako, Sant Just Desvern, Spain). Immunofluorescence images were acquired using standard filter sets using an Olympus FluoView TM FV1000 confocal microscope and the FV10_ASW 4.2 visualization software.

α-SYN fluorescence intensity for each condition was analyzed in an average of 150 cells from 6 different random fields at an objective magnification of 40×. Quantification of Thioflavin S positive aggregates in each condition was analyzed in an average of 20 cells from 3 different random fields by measuring the fluorescent area per cell. All quantification analyses were done using ImageJ software (NIH, USA).

### Cell Culture Experiments with iPSC Cell Lines Derived from Human Patients

Previously generated iPSC lines SP-11.1 (from control) and SP-12.3 (from patients with familial PD with the LRRK2 G2019S mutation) were used and culture and differentiation were carried out as described, (34), following a protocol approved by the Spanish competent authorities (Commission on Guarantees concerning the Donation and Use of Human Tissues and Cells of the Carlos III Health Institute). Briefly, hiPSC were cultured on matrigel (Corning Limited, Life Sciences, UK) and maintained in hESC medium, consisting on KO-DMEM (Invitrogen, Thermo Fisher Scientific, Madrid, Spain) supplemented with 20% KO-Serum Replacement (Invitrogen, Thermo Fisher Scientific, Madrid, Spain), 2 mM Glutamax (Invitrogen, Thermo Fisher Scientific, Madrid, Spain), 50 μM 2-mercaptoethanol (Invitrogen, Thermo Fisher Scientific, Madrid, Spain), non-essential aminoacids (Cambrex, Nottingham, UK) and 10 ng/ml bFGF (Peprotech, London, UK). Medium was preconditioned overnight by irradiated mouse embryonic fibroblast and hiPSC were maintained on Matrigel (Corning Limited, Life Sciences, UK) at 37 °C, 5% CO2.

For DAn differentiation, iPSC were transduced with LV.NES.LMX1A.GFP and processed as previously described (79). Briefly, confluent iPSC 10 cm dishes were disaggregated with accutase and embryoid bodies (EB) were generated using forced aggregation method in V-shaped 96 well plates. Two days later, EBs were patterned as ventral midbrain by culturing them in suspension for 10 days in N2B27 supplemented with 100 ng/ml SHH (Peprotech, London, UK), 100 ng/ml FGF8 (Peprotech, London, UK) and 10 ng/ml FGF2 (Peprotech, London, UK). Then, for α-SYN and neurite analysis, differentiation to midbrain DAn was performed on the top of PA6 murine stromal cells for 3 weeks (79). To analyze **α**-SYN levels, neuronal cultures were gently trypsinized and re-plated in matrigel-coated slides for 3 days, then transduced with the two different RAC1 isoforms or the control LV, at an estimated MOI of 10. Cells were fixed and analyzed 7 days after transduction. For long-term culture studies, EBs were mechanically dissociated by repeated pipetting after the induction step. Salient EB fragments were transduced with the two different RAC1 isoforms or the control LV at an estimated MOI of 3. Three days post-transduction, the aggregates were seeded on primary murine astrocytes and cultured for 9 weeks.

#### Lentiviral Vectors

RAC1 wild type (WT) and constitutively active (V12) (CA) forms were amplified by means of PCR from expression plasmids kindly provided by (Dr Francisco Sánchez-Madrid; Spanish National Center for Cardiovascular Research (CNIC), Madrid, Spain). Subsequently, RAC1-(WT) and RAC1-(CA) cDNA were cloned under the human phosphoglycerate kinase (PGK) promoter of pCCL.cPPT-hPGK-IRES.eGFP-WPRE lentiviral transfer vector. High-titer vesicular stomatitis virus (VSV)–pseudotyped LV stocks were produced in 293T cells by calcium phosphate–mediated transient transfection of the transfer vector, the late generation packaging construct pMDL, and the VSV envelope–expressing construct pMD2.G, and purified by ultracentrifugation as previously described [87]. Expression titers, determined on HeLa cells by fluorescence-activated cell sorter (FACS) analysis (FACSCalibur, Becton Dickinson), were as follows: LV-RAC1 (WT): 2.60 · 10^8 TU/mL; LV-RAC1 (CA): 2.24 · 10^8 TU/mL; LV-PGK-GFP: 5.08 · 10^9 TU/mL.

#### Immunofluorescence for iPSC-Derived Cells

iPSC-derived cells were fixed with 4% paraformaldehyde (PFA) in Tris-buffered saline (TBS) buffer for 20 min and blocked in 0.3% Triton X-100 (Sigma-Aldrich, Madrid, Spain) with 3% donkey serum for 2 h. In the case of α-SYN and LC3 staining, Triton X-100 was kept at 0.01% for the blocking and antibody incubation steps. The following primary antibodies were used: mouse anti-α-SYN (610787; 1:500) (BD Biosciences, Madrid, Spain), rabbit anti-TH (sc-14007; 1:500) (Santa Cruz Biotechnology, Madrid, Spain), chicken anti-GFP (1020; 1:250) from Aves Labs (Cosmo Bio, AbBcn S.L., Bellaterra, Spain), rabbit anti-cleaved Caspase3 (9664; 1:400) (Cell Signaling) and LC3B (2775; 1:100) (Cell Signaling). Images were acquired using a Leica SP5 confocal microscope.

#### α-SYN and Neurite Analysis

α-SYN and neurite analysis were performed after a total of 4 weeks of differentiation plus 1 week after LV transduction on iPSC-derived DA neurons. DA neurons were randomly selected, using a Leica SP5 confocal microscope, and analyzed with ImageJ for α-SYN intensity levels and with the ImageJ plugin NeuronJ to determine the number of neurites per cell, number of terminals and branch points.

#### Cleaved Caspase-3, Apoptotic Cell Number and Autophagosome Accumulation and Analysis

Cleaved caspase-3, apoptotic cell number and autophagosome accumulation analysis were performed after a total of 9 weeks of differentiation on iPSC-derived DAn grown on murine astrocytes. DA neurons were randomly selected, using a Leica SP5 confocal microscope, and analyzed with ImageJ to determine the fraction of the neuronal soma area stained by cleaved caspase-3 or covered by LC3-positive particles.

#### Statistical Analysis

All experiments were performed at least three independent times. All data are presented as mean ± SEM. Group means were compared with either the Student’s *t* test or ANOVA. All *P* values were two tailed, and a *P* value of less than 0.05 was considered statistically significant. All statistical analyses were analyzed using GraphPad Prism (San Diego, CA, USA) software.

Outlier values were identified with the Grubbs’ tests and excluded from the analysis.

Differences among means were analyzed either by 1- or 2-way analysis of variance (ANOVA), as appropriate, using the post hoc Tukey’s multiple comparison test. In all cases, the null hypothesis was rejected at the 0.05 level.

### Biological Enrichment Analysis

Transcriptomic analysis of iPSC-derived DAn from PD patients (*n* = 10) and healthy controls (*n* = 4) was done as previously described [54]. Identified differentially expressed genes (DEGs) after multiple testing adjustment of *P* values (*n* = 437) were subjected to biological enrichment analysis. To this end, we used the Core Analysis module of the Ingenuity® Pathway Analysis (IPA) software (Qiagen, Redwood City, www.qiagen.com/ingenuity) to identify biological enrichment of canonical pathways deregulated in iPSC-derived DAn from PD patients. More specifically, we used the Ingenuity Knowledge Base of Genes, and considered only direct molecules and/or relationships for humans. Statistical significance of canonical pathways was computed by using the Fischer’s exact test and significance levels were set at P below 0.05. Using IPA we also calculated Z-score values which consider the directional effect of one molecule on another molecule or on a process, and also the direction of change of molecules in the dataset and provide predictions about whether the pathway is predicted to be activated or inhibited, or if the pathway is ineligible for such an assessment.

## Electronic supplementary material

ESM 1 (PDF 421 kb)

ESM 2 (PDF 200 kb)

Video 1 (AVI 256315 kb)
