# Supplementary material for: The Small GTPase RAC1/CED-10 Is Essential in Maintaining Dopaminergic Neuron Function and Survival Against α-Synuclein-Induced Toxicity
Source: Mol Neurobiol. 2018 Feb 10;55(9):7533–52. doi: 10.1007/s12035-018-0881-7 (PMC6096980; doi:10.1007/s12035-018-0881-7)
Supplement: Supplementary file 1 — (PDF 421 kb) [file 12035_2018_881_MOESM1_ESM.pdf]

Online Resources

The small GTPase RAC1/CED-10 is essential in maintaining dopaminergic neuron function and survival against α-synuclein-induced toxicity

Hanna Kim[1] (*), Carles Calatayud-Aristoy[2,3] (*), Sangib K Guha[4] (*), Irene Fernández-Carassa[2,3], Laura Berkowitz[1], Iria Carballo-Carbajal[5], Mario Ezquerra[6], Rubén Fernández-Santiago[6], Pankaj Kapahi[4], Ángel Raya[7], Antonio Miranda-Vizuete[8], Jose Miguel Lizcano[9], Miquel Vila [5,9,10], Kimberlee Caldwell [1], Guy A Caldwell [1], Antonella Consiglio[2,3] and Esther Dalfo (**) [9].

(1) Department of Biological Sciences, The University of Alabama, Tuscaloosa, Alabama 35487, USA.

(2) Institute for Biomedicine (IBUB) University of Barcelona (UB), Barcelona 08028, Spain.

(3) Department of Pathology and Experimental Therapeutics, School of Medicine, University of Barcelona, 08908 Barcelona, Spain

(4) Buck Institute for Research on Aging, 8001 Redwood Boulevard, Novato, CA 94945, USA

(5) Neurodegenerative Diseases Research Group, Vall d'Hebron Research Institute-Center for Networked Biomedical Research on Neurodegenerative Diseases (CIBERNED), 08035 Barcelona, Spain.

(6) Laboratory of Parkinson Disease and Other Neurodegenerative Movement Disorders, Department of Neurology: Clinical and Experimental Research, IDIBAPS – Hospital Clínic de Barcelona, 08036, Barcelona, Spain

(7) Center of Regenerative Medicine in Barcelona (CMRB), Hospital Duran i Reynals, 3rd Floor, Av. Gran Via 199-203, 08908 Hospitalet de Llobregat (Barcelona), Spain

(8) Instituto de Biomedicina de Sevilla, Hospital Universitario Virgen del Rocío/CSIC/ Universidad de Sevilla, Sevilla 41013, Spain.

(9) Department of Biochemistry and Molecular Biology, Institut de Neurociències, Faculty of Medicine, M2 , Universitat Autònoma de Barcelona (UAB), Bellaterra Campus , Cerdanyola del Vallés, Barcelona, Spain. ORCID: 0000-0003-4677-8515

(10) Catalan Institution for Research and Advanced Studies (ICREA), 08010 Barcelona, Spain

(**) Corresponding author: esther.dalfo@uab.cat +34935811624//+34676136881

Online Resource 1: (A) Representative RNAi empty vector (EV) fed worms expressing GFP and α-SYN specifically in dopaminergic neurons (P*dat-1*:: α-SYN + P*dat-1*:: GFP) at the different time points indicated (L4 +7, L4 +3 and L4 +1) and fed with empty vector (EV) or *ced-10* RNAi clones. Filled white arrowhead labels healthy neurons whereas degenerated or missing neurons are labeled with an open arrow. Magnification bar is 30 µm **(B)** Percentage of P*dat-1*:: α-SYN + P*dat-1*:: GFP worms non- depleted and *ced-10* depleted by RNAi, that had the full complement of six anterior dopaminergic neurons at the different time points indicated. Data are mean ± SEM. Statistics were obtained by comparing *ced-10* RNAi depleted worms. Statistics: Student's *t*-test *** $P < 0.001$. Number of animals is 30-35 per condition, and the experiment was repeated 3 times independently.

Online Resource 2: Representative movie of the thrashing assay performed in a N2 wild type animal at L4 + 5 days of development.

Online Resource 3: Representative movie of the thrashing assay performed in a *pkIs2386* [P*unc-54::*α-SYN*::*YFP] animal at L4 + 5 days of age.

Online Resource 4: Representative movie of the thrashing assay performed in a *pkIs2386* [P*unc-54*::α-SYN::YFP *; ced-10(n3246)*] at L4 + 5 days of age.

Online Resource 5: Characterization of the α-SYN aggregation model in neuroblastoma cells. (A) Representative confocal images of BE(2)-M17 cells over-expressing α-SYN and differentiated with 10 µM retinoic acid (RA) for 10 days. Immunofluorescence against α-SYN with mouse monoclonal (upper row) and rabbit

polyclonal (lower row) antibodies in cells untreated (first panel) or treated with the histone deacetylase inhibitor sodium butyrate (SB) at a concentration of 10 mM for 36 h. White arrows highlight α-SYN aggregates in soma and neurites. **(B)** Quantification of the average α-SYN fluorescence intensity in individual SB-treated or untreated cells. Data are presented as mean ± SEM. N = 215 (UT) and N = 70 (SB), from at least 3 independent experiments. Statistics T-test with Welch's correction *** P<0.0001. **(C)** Representative confocal micrographs of differentiated α-syn over-expressing BE(2)-M17 cells and treated with SB. Cells were transduced with Control-GFP (upper row), RAC1 (WT)-GFP (middle row) or RAC1 (CA)-GFP (bottom row) and subjected to immunofluorescence for RAC1 (green) and α-SYN (red). **(D)** Quantification of the average α-SYN fluorescence intensity in individual cells transduced with (WT)- or (CA)-RAC1 compared to Control-GFP. Data are presented as mean ± SEM. N = 128 (EV), N = 169 (WT) and N = 159 (CA), from at least 3 independent experiments. One-way ANOVA followed by Dunnet's post hoc test * P <0.05 and ** P<0.01. Scale bars represent 10 μm.

Online Resource 6: Methodology to get dopaminergic neurons from fibroblasts derived from LRRK2-PD patients (A) Scheme depicting the *in vitro* dopaminergic neuron differentiation protocol for short-term studies. Embryoid bodies (EBs) generated by forced aggregation of iPSC colonies were patterned to ventral midbrain in the presence of N2B27 medium supplemented with specific morphogenesis for 10 days. Neutralized EBs were then seeded on a confluent monolayer of PA6 stromal cells and they were let to differentiate for up to 21 days before disaggregation and subsequent LV-RAC1 transduction. Transduced DAn were analyzed 3 days post infection (d.p.i.) **(B)** Scheme depicting the *in vitro* DAn differentiation protocol for long-term studies. EBs generated by forced aggregation of iPSC colonies were patterned to ventral midbrain in the presence of N2B27 medium supplemented with specific morphogenesis for 10 days. Neutralized EBs were gently disaggregated to small clumps and transduced with

LV-RAC1 or LV-GFP. 3 d.p.i.,transduced EBs were then seeded on a confluent monolayer of murine cortical astrocytes and they were let to differentiate for up to 75 days before they were processed for analysis. **(C)** Immunofluorescence analysis of dopaminergic neurons differentiated from different iPSC lines and cultured for 21 days on the top of PA6 stromal cells and transduced with the different LV constructs. Cells were co-stained for TH (red), GFP (green) and the nuclei were counterstained with DAPI (blue) **(D)** Scheme depicting the polycistronic LV construct engineered for overexpressing RAC1 WT or CA forms along with GFP **(E)** Western blot analysis of Rac1 levels in 293T cells transduced with LV RAC1 (WT), LV-RAC1 (CA) and LV-GFP as control.β-actin was used as loading control.

Online Resource 7: Representative analysis of neuronal arborisation obtained by ImageJ plug-in NeuronJ; Primary neurites are traced in red, secondary neurites are traced in green and tertiary neurites are traced in blue. Magnification bar is 10 μm.

Online Resource 8: Analysis of canonical pathways affected in iPSC-derived dopaminergic neurons from PD. The analysis was performed selecting differentially expressed genes from the study of Fernández-Santiago et al 2015 [1] and analyzing the data on the Core Analysis from Ingenuity Pathways Analysis (IPA) software. We used the General settings for IPA system as follows: «Ingenuity® Knowledge Base (genes)», «considered only molecules and/or relationships for humans». Statistical significance was measured by Fischer's exact test to calculate a *P* value (in a logarithmic scale shown as bars). *P* value of ≤0.05 is showed with a line threshold. The ratio represents the number of differentially expressed genes in a given pathway divided by total number of genes that make up that canonical pathway (shown as squares). The related signaling Rho/GTPases, axonal guidance and Wnt signaling pathways were specifically altered.

Online Resource 9: List of differentially expressed genes (DEGs) from the signaling by Rho family GTPases pathway showing altered expression in iPSC-derived

dopaminergic neurons from PD patients compared to controls. The differential expression data is presented as log2 ratio (Expr log ratio).

**Symbol**	**Entrez Gene Name**	**Expr Log Ratio**	**Location**	**Type**	**Entrez Gene ID for Human**
ACTG2	actin, gamma 2, smooth muscle, enteric	-1.685	cytoplasm	other	72
CDC42EP3	CDC42 effector protein 3	-0.734	cytoplasm	other	10602
CDH4	cadherin 4	0.878	plasma membrane	other	1002
CDH5	cadherin 5	-1.239	plasma membrane	other	1003
CDH6	cadherin 6	1.302	plasma membrane	other	1004
CDH20	cadherin 20	1.43	plasma membrane	other	28316
GNAL	G protein subunit alpha L	0.669	cytoplasm	enzyme	2774
GNAZ	G protein subunit alpha z	0.689	plasma membrane	enzyme	2781
GNG3	G protein subunit gamma 3	2.334	plasma membrane	enzyme	2785
GNG11	G protein subunit gamma 11	-1.707	plasma membrane	enzyme	2791
ITGA3	integrin subunit alpha 3	-0.645	plasma membrane	other	3675
MAPK10	mitogen-activated protein kinase 10	1.243	cytoplasm	kinase	5602
NEDD4	neural precursor cell expressed, developmentally down-regulated 4, E3 ubiquitin protein ligase	-0.692	cytoplasm	enzyme	4734
PAK3	p21 (RAC1) activated kinase 3	1.246	cytoplasm	kinase	5063
PLD1	phospholipase D1	-0,91	cytoplasm	enzyme	5337
WASF3	WAS protein family member 3	0.932	cytoplasm	other	10810

References

1. Fernández-Santiago, R., et al., *Aberrant epigenome in iPSC-derived dopaminergic neurons from Parkinson's disease patients.* EMBO Mol Med, 2015. **7**(12): p. 1529-46.
