# Supplementary material for: The Small GTPase RAC1/CED-10 Is Essential in Maintaining Dopaminergic Neuron Function and Survival Against α-Synuclein-Induced Toxicity
Source: Mol Neurobiol. 2018 Feb 10;55(9):7533–52. doi: 10.1007/s12035-018-0881-7 (PMC6096980; doi:10.1007/s12035-018-0881-7)

Online Resource 1

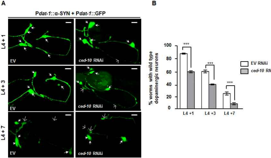

Online Resource 5

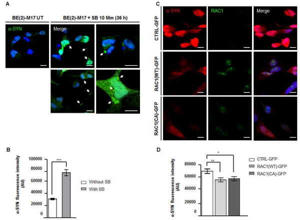

Online Resource 6

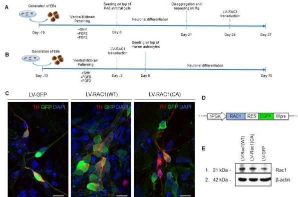

Online Resource 7

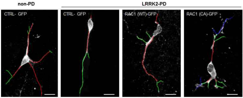

Online Resource 8

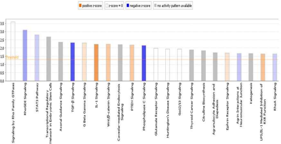

Supplement: Supplementary file 2 — (PDF 200 kb) [file 12035_2018_881_MOESM2_ESM.pdf]
